# Epigenetic measures of ageing predict the prevalence and incidence of leading causes of death and disease burden

**DOI:** 10.1186/s13148-020-00905-6

**Published:** 2020-07-31

**Authors:** Robert F. Hillary, Anna J. Stevenson, Daniel L. McCartney, Archie Campbell, Rosie M. Walker, David M. Howard, Craig W. Ritchie, Steve Horvath, Caroline Hayward, Andrew M. McIntosh, David J. Porteous, Ian J. Deary, Kathryn L. Evans, Riccardo E. Marioni

**Affiliations:** 1 Centre for Genomic and Experimental Medicine, Institute of Genetics and Molecular Medicine, University of Edinburgh, Edinburgh, EH4 2XU UK; 2 Institute of Psychiatry, Psychology and Neuroscience, King’s College London, London, SE5 8AF UK; 3 Division of Psychiatry, Centre for Clinical Brain Sciences, University of Edinburgh, Edinburgh, EH16 4UX UK; 4 Edinburgh Dementia Prevention, Centre for Clinical Brain Sciences, University of Edinburgh, Edinburgh, EH16 4UX UK; 5 Department of Human Genetics, David Geffen School of Medicine, University of California Los Angeles, Los Angeles, 90095-7088 USA; 6 Department of Biostatistics, Fielding School of Public Health, University of California Los Angeles, Los Angeles, 90095-1772 USA; 7 MRC Human Genetics Unit, Institute of Genetics and Molecular Medicine, University of Edinburgh, Edinburgh, EH4 2XU UK; 8 Lothian Birth Cohorts, University of Edinburgh, Edinburgh, EH8 9JZ UK

**Keywords:** DNA methylation, Biological ageing, Epigenetic age acceleration, Epidemiology

## Abstract

**Background:**

Individuals of the same chronological age display different rates of biological ageing. A number of measures of biological age have been proposed which harness age-related changes in DNA methylation profiles. These measures include five ‘epigenetic clocks’ which provide an index of how much an individual’s biological age differs from their chronological age at the time of measurement. The five clocks encompass methylation-based predictors of chronological age (HorvathAge, HannumAge), all-cause mortality (DNAm PhenoAge, DNAm GrimAge) and telomere length (DNAm Telomere Length). A sixth epigenetic measure of ageing differs from these clocks in that it acts as a speedometer providing a single time-point measurement of the pace of an individual’s biological ageing. This measure of ageing is termed DunedinPoAm. In this study, we test the association between these six epigenetic measures of ageing and the prevalence and incidence of the leading causes of disease burden and mortality in high-income countries (*n* ≤ 9537, Generation Scotland: Scottish Family Health Study).

**Results:**

DNAm GrimAge predicted incidence of clinically diagnosed chronic obstructive pulmonary disease (COPD), type 2 diabetes and ischemic heart disease after 13 years of follow-up (hazard ratios = 2.22, 1.52 and 1.41, respectively). DunedinPoAm predicted the incidence of COPD and lung cancer (hazard ratios = 2.02 and 1.45, respectively). DNAm PhenoAge predicted incidence of type 2 diabetes (hazard ratio = 1.54). DNAm Telomere Length associated with the incidence of ischemic heart disease (hazard ratio = 0.80). DNAm GrimAge associated with all-cause mortality, the prevalence of COPD and spirometry measures at the study baseline. These associations were present after adjusting for possible confounding risk factors including alcohol consumption, body mass index, deprivation, education and tobacco smoking and surpassed stringent Bonferroni-corrected significance thresholds.

**Conclusions:**

Our data suggest that epigenetic measures of ageing may have utility in clinical settings to complement gold-standard methods for disease assessment and management.

## Background

The sustained increase in global life expectancy and population size has prompted a concomitant elevation in the prevalence of chronic disease states [1]. The World Health Organisation specifies ten leading causes of mortality and ten leading causes of disease burden. In high-income countries, six diseases are present in both sets: ischemic heart disease, stroke, lung cancer, Alzheimer’s disease (AD) and other dementias, diabetes and chronic obstructive pulmonary disease (COPD). The remaining four leading causes of mortality are lower respiratory tract diseases, bowel cancer, kidney disease and breast cancer [2]. The additional four causes of disease burden are back or neck pain, skin disease, sense organ disease and depression [3]. Many of these disease states encompass heterogeneous, complex aetiologies resulting in a paucity of effective treatment paradigms. Given the number of individuals affected by such disorders and the associated burden, there is an urgent need for effective molecular predictors in clinical settings that can identify individuals on trajectories towards disease.

Ageing is a major risk factor for many common disease states. However, individuals of the same chronological age exhibit disparate rates of biological ageing and susceptibilities to common morbidities and mortality. Differential patterns of biological ageing among individuals may be exploited to identify novel predictors of disease [4]. Recently, a number of strategies have been proposed to estimate biological age by leveraging inter-individual variation in DNA methylation (DNAm) profiles. These epigenetic measures of ageing, many of which are called ‘epigenetic clocks’, correlate strongly with chronological age [5]. Moreover, for a given chronological age, an accelerated epigenetic age or faster rate of ageing is associated with an increased risk of mortality and shows cross-sectional relationships with age-related morbidities [6–10].

In this paper, we focus on six epigenetic predictors of ageing. In 2013, Horvath developed a pan-tissue epigenetic clock, termed ‘Horvath Age’, derived from the linear combination of 353 CpG sites in multiple tissues [11]. Hannum created a DNAm-based clock termed ‘Hannum Age’ based on 71 CpG sites in blood tissue [12]. Levine et al. proposed a predictor of lifespan and health by developing a methylation-based predictor of an individual’s ‘phenotypic age’ (‘DNAm PhenoAge’). Phenotypic age is informed by chronological age as well as haematological and biochemical measures, including creatinine levels and lymphocyte percent [13]. Lu et al. proposed ‘DNAm GrimAge’ as a predictor of mortality and demonstrated that it outperforms existing clocks in predicting death and age-related conditions, including cardiovascular disease [14]. DNAm GrimAge was developed in two stages. In the first stage, DNAm-based surrogates for 88 plasma protein levels and smoking pack years were developed using elastic net regression models. Only DNAm-based surrogates which exhibited a correlation coefficient of at least 0.35 with their respective phenotype were considered for stage two. In addition to smoking pack years, 12/88 protein proxies satisfied this condition. In the second stage, time-to-death due to all-cause mortality was regressed on chronological age, sex, DNAm-based surrogates for smoking pack years and 12 plasma protein levels. The model selected chronological age, sex, DNAm-based proxies for smoking pack years and the levels of 7/12 plasma proteins; the linear combination of these variables provides a measure of DNAm GrimAge. Furthermore, telomere length is associated with cardiovascular disease, cancer risk and all-cause mortality [15–17]. Lu et al. proposed a DNAm-based estimator of telomere length termed ‘DNAm Telomere Length’ (DNAm TL) which exhibits stronger associations with lifespan, smoking history and body mass index when compared to phenotypic telomere length as measured by quantitative polymerase chain reaction or Southern blotting [18]. These five measures of biological ageing provide an index of the difference between an individual’s biological age and chronological age at the time of measurement and are derived from cross-sectional measurements across individuals of varying ages. This can approximate a longitudinal ageing trajectory but can also be confounded by the possibility that individuals who were born in different years may have been exposed to different early-life exposures [4]. As a result, individuals of different chronological ages may display differential DNAm patterns that do not reflect age-related DNAm changes but rather reflect differential early-life environmental influences. To address this, Belsky et al. (2015) developed a longitudinal measure of biological age by examining the rate of change in 18 blood-chemistry and organ-system-function biomarkers at three successive time points from ages 26 to 38 in participants of the Dunedin study (*n* = 954) [19]. This measure was termed ‘Pace of Aging’ (PoA), and all of the individuals in the sample were born in 1972–1973. Recently, Belsky et al. (2020) derived a DNAm-based proxy of PoA termed ‘DunedinPoAm’ [20]. The authors state that this measure reflects a speedometer which tracks how fast the subject is ageing whereas the previous measures of epigenetic ageing represent clocks recording how much time has passed.

For epigenetic clocks, the difference between an individual’s methylation-based age and their chronological age provides a measure of accelerated or decelerated ageing. DunedinPoAm returns a measure in years of biological ageing per each calendar year with higher values reflecting a faster rate of ageing. Higher values of DunedinPoAm as well as age-adjusted Horvath Age, Hannum Age, DNAm PhenoAge and DNAm GrimAge are hypothesised to associate with poorer health outcomes as these measures capture accelerated biological ageing. Lower values of age-adjusted DNAm TL are hypothesised to correlate with poorer health as this reflects shorter telomere length. To date, a number of studies have demonstrated associations between epigenetic measures of ageing and risk of mortality and disease states [21–24] or have provided comparisons of such epigenetic measures [25–30]. However, no study has compared all six epigenetic measures of ageing with respect to their association with a broad range of common health conditions.

In this study, we test the association between the six epigenetic measures of ageing and the prevalence*,* and incidence*,* of the ten leading causes of mortality and disease burden (as indexed by disability-adjusted life years; DALYs) [2, 3]. In addition, we examine their association with continuous traits underlying these conditions, such as lung function tests for chronic obstructive pulmonary disease (COPD). We utilise DNA methylation array data and electronic health record data from a Scottish cohort, Generation Scotland: Scottish Family Health Study (GS:SFHS or GS). GS is a family-based cohort consisting of over 20,000 individuals with rich health and lifestyle information. Genome-wide methylation data were generated on approximately 10,000 participants making it one of the largest DNAm resources in the world. We examine associations between epigenetic measures of ageing and prevalent disease as well as an assessment of their ability to predict time-to-disease onset. These findings may expedite the future use and refinement of large-scale molecular data-based approaches for predicting clinically defined outcomes and subsequent individual disease risk prediction.

## Results

### Demographics and epigenetic measures of ageing

In the discovery cohort, 56.3% of the participants were female with a mean age of 51.4 years (standard deviation (SD) = 13.2) (*n* = 4450). The mean values for epigenetic measures of ageing were as follows: Horvath age (60.1 years, SD = 9.8), Hannum age (47.4 years, SD = 9.6), DNAm PhenoAge (43.7 years, SD = 11.5), DNAm GrimAge (48.8 years, SD = 10.9), DNAm Telomere Length (7.4 kilobase pairs, SD = 0.3), and DunedinPoAm (1.1 years of biological ageing per each calendar year, SD = 0.1). Summary data for all variables in this study are presented in Additional file 1.

In the replication cohort, 61.4% were female with a mean age of 50.0 years (SD = 12.5) (*n* = 2578). Values for all phenotypes were comparable between discovery and replication cohorts with the exception of DNAm GrimAge (discovery: 48.8 years, SD = 10.9, replication: 60.5 years, SD = 10.6), and the incidence of self-reported depression (discovery: 8.4%, replication: 16.4%), and SCID (Structured Clinical Interview for DSM)-identified Depression (discovery: 18.5%, replication: 38.2%). This disparity in depression prevalence reflects an over-sampling of depression cases in the replication cohort. It is unclear as to why the replication cohort shows a higher mean DNAm GrimAge. However, it is possible that this difference may be driven by a latent aspect of poorer overall health that may be associated or correlated with depression.

### Epigenetic measures of ageing and disease prevalence

In a basic model adjusting for age and sex, 51 phenotypes were significant at Bonferroni-corrected levels of significance in both the discovery and replication cohorts (Additional file 2: Note 1 and Additional file 3: Tables S1-S4). In the discovery cohort, a Bonferroni-corrected threshold of *P* < 2.54 × 10^-4^ was applied as this corrected for all tests performed (0.05/197 tests). Of these 197 models, 78 were significant at *P* < 2.54 × 10^-4^ in the discovery set and were therefore carried forward to the replication stage. In the replication set, associations which surpassed a Bonferroni-corrected threshold of *P* < 6.41 × 10^-4^ were deemed significant (0.05/78 tests). Additional file 4: Fig. S1-S3 highlight significant associations present in both sets for categorical traits, continuous traits and all-cause mortality, respectively. A measure-by-measure comparison of associations with categorical and continuous phenotypes from fully adjusted models in the replication cohort, stratified by disease type, is shown in Additional file 5. For all models, beta coefficients for continuous traits were correlated 0.96 between discovery and replication sets. For categorical phenotypes, the correlation coefficient for log odds was 0.79 between sets (Additional file 4: Fig. S4).

**Fig. 1:**
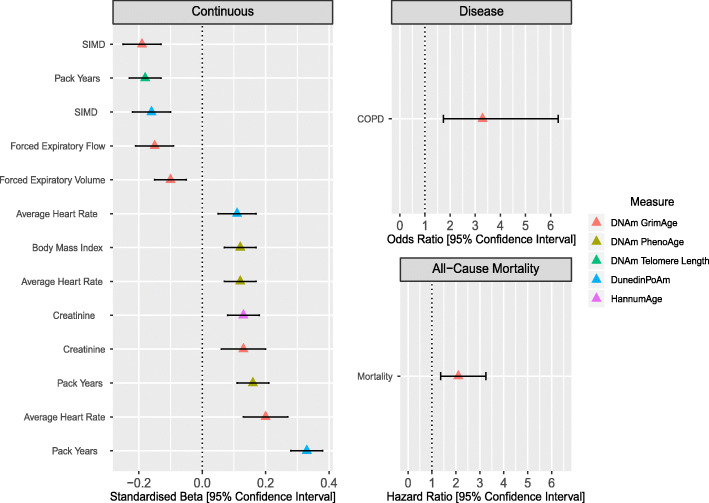
The associations between epigenetic measures of ageing and disease prevalence, continuous traits and all-cause mortality in Generation Scotland. Only associations present in discovery and replication sets are shown, and replication test statistics are presented. *Continuous:* Age-adjusted DNAm GrimAge was associated with greater deprivation (lower SIMD rank), reduced forced expiratory flow and forced expiratory volume. Age-adjusted DNAm GrimAge was positively associated with serum creatinine levels and average heart rate. Age-adjusted DNAm PhenoAge was positively associated with body mass index, average heart rate and smoking pack years. Age-adjusted DNAm Telomere Length was negatively associated with smoking pack years. Higher values for DunedinPoAm were associated with greater deprivation (lower SIMD rank), a higher average heart rate and smoking pack years. Age-adjusted Hannum Age was positively associated with serum creatinine levels. *Disease:* Age-adjusted DNAm GrimAge alone was associated with the prevalence of COPD in both discovery and replication sub-cohorts after correction for multiple testing. *All-Cause Mortality:* Age-adjusted DNAm GrimAge alone was associated with all-cause mortality in both sets after multiple testing correction. Associations represent a one standard deviation increase in the respective measure of biological ageing. Models were adjusted for age, sex, alcohol consumption, body mass index, deprivation, education and smoking. Models involving lung function tests were also corrected for height. COPD (chronic obstructive pulmonary disease), SIMD (Scottish Index of Multiple Deprivation)

Fifteen relationships remained significant in both discovery and replication sets in a fully adjusted model accounting for age, sex and five common risk factors (Additional file 3: Tables S5 and S6, respectively). Those relationships which were significant in both cohorts at a Bonferroni-corrected significance threshold of *P* < 6.41 × 10^-4^ (reflecting the same stringent threshold as above) are reported herein and presented in Table 1 and Fig. 1.

**Table 1:** Significant and replicated relationships between epigenetic age measures and prevalent disease data, and continuous traits

		Discovery cohort			Replication cohort		
*Categorical phenotypes*							
Measure	Variable	*n* event	OR	*P*	*n* event	OR	*P*
DNAm GrimAge	COPD	48	2.00	1.0 × 10^−4^	32	3.29	3.4 × 10^−4^
*Continuous phenotypes*							
Measure	Variable	*n*	*β*	*P*	*n*	*β*	*P*
DunedinPoAm	Pack Years	2419	0.45	1.2 × 10^−112^	1340	0.33	7.6 × 10^−36^
DNAm TL	Pack Years	2419	-0.14	1.1 × 10^−11^	1340	-0.18	2.7 × 10^−11^
DNAm PhenoAge	Pack Years	2419	0.11	3.0 × 10^−08^	1340	0.16	9.5 × 10^−10^
DNAm GrimAge	SIMD	2419	-0.13	5.9 × 10^−08^	1340	-0.19	4.6 × 10^−09^
DNAm GrimAge	Average Heart Rate	2416	0.19	1.4 × 10^−12^	1339	0.20	1.6 × 10^−08^
DunedinPoAm	SIMD	2419	-0.13	1.8 × 10^−09^	1340	-0.16	2.2 × 10^−08^
Hannum Age	Creatinine	2406	0.21	1.4 × 10^−26^	1334	0.13	4.2 × 10^−07^
DNAm GrimAge	FEF	2055	-0.12	1.2 × 10^−06^	1149	-0.15	1.4 × 10^−06^
DNAm PhenoAge	Body Mass Index	2419	0.12	2.5 × 10^−10^	1340	0.12	7.4 × 10^−06^
DNAm PhenoAge	Average Heart Rate	2416	0.11	2.1 × 10^−07^	1339	0.12	7.7 × 10^−06^
DNAm GrimAge	FEV	2074	-0.08	2.0 × 10^−05^	1151	-0.10	1.4 × 10^−04^
DNAm GrimAge	Creatinine	2406	0.19	3.0 × 10^−15^	1334	0.13	2.0 × 10^−04^
DunedinPoAm	Average Heart Rate	2416	0.19	1.6 × 10^−15^	1339	0.11	2.2 × 10^−04^
*Mortality analysis*							
Measure	Variable	*n* event	HR	*P*	*n* events	HR	*P*
DNAm GrimAge	All-cause mortality	89	1.62	1.4 × 10^−4^	30	2.10	5.6 × 10^−4^

Analyses were performed using a fully adjusted model accounting for age, sex, alcohol consumption, body mass index, deprivation, education and smoking pack years

*COPD* chronic obstructive pulmonary disease, *FEF* forced expiratory flow, *FEV* forced expiratory volume, *HR* hazard ratio, *OR* odds ratio, *SIMD* Scottish Index of Multiple Deprivation, *TL* telomere length

### Associations with disease

In relation to prevalent disease data, only the association between an accelerated DNAm GrimAge and COPD remained significant in both cohorts in the fully adjusted model (replication cohort: odds ratio (OR) per SD = 3.29, 95% confidence interval (CI) = [1.73, 6.30], *P* = 3.4 × 10^-4^; Fig. 1)

### Associations with all-cause mortality

An accelerated DNAm GrimAge alone was associated with all-cause mortality following adjustment for the lifestyle risk factors (replication cohort: hazard ratio (HR) per SD = 2.10, 95% CI = [1.36, 3.25], *P* = 5.6 × 10^-4^; Fig. 1).

### Associations with continuous clinically associated traits

An accelerated DNAm GrimAge was associated with greater deprivation (a lower Scottish Index of Multiple Deprivation (SIMD) rank; *β*_replication_ = -0.19, 95% CI = [-0.25, -0.13], *P* = 4.6 × 10^−9^), an increased average heart rate (*β*_replication_ = 0.20, 95% CI = [0.13, 0.27], *P* = 1.6 × 10^−8^), a reduced forced expiratory flow (*β*_replication_ = -0.15, 95% CI = [-0.21, -0.09], *P* = 1.4 × 10^−6^), a reduced forced expiratory volume (*β*_replication_ = -0.10, 95% CI = [-0.15, -0.05], *P* = 1.4 × 10^−4^) and increased serum creatinine levels (*β*_replication_ = 0.13, 95% CI = [0.06, 0.20], *P* = 2.0 × 10^−4^).

Higher values of DunedinPoAm, indicating a faster rate of ageing, were positively associated with smoking pack years (*β*_replication_ = 0.33, 95% CI = [0.28, 0.38], *P* = 7.6 × 10^−36^), greater deprivation (lower SIMD rank; *β*_replication_ = -0.16, 95% CI = [-0.22, -0.10], *P* = 2.2 × 10^−8^) and average heart rate (*β*_replication_ = 0.11, 95% CI = [0.05, 0.17], *P* = 2.2 × 10^−4^).

An accelerated DNAm PhenoAge was associated with smoking pack years (*β*_replication_ = 0.16, 95% CI = [0.11, 0.21], *P* = 9.5 × 10^−10^), an increased body mass index (*β*_replication_ = 0.12, 95% CI = [0.07, 0.17], *P* = 7.4 × 10^−6^) and an increased average heart rate (*β*_replication_ = 0.12, 95% CI = [0.07, 0.17], *P* = 7.7 × 10^−6^).

Age-adjusted DNAm Telomere Length was negatively associated with smoking pack years (*β*_replication_ = -0.18, 95% CI = [-0.23, -0.13], *P* = 2.7 × 10^−11^). An accelerated DNAm Hannum Age (EEAA) was associated with increased serum creatinine levels (*β*_replication_ = 0.13, 95% CI = [0.08, 0.18], *P* = 4.2 × 10^−7^).

### Covariate-specific attenuation

To examine the contribution of each of the five common disease risk factors in attenuating the 51 significant associations brought forward to the fully adjusted model, we repeated each model including only one of these five covariates at a time. These risk factors were alcohol consumption, body mass index, deprivation, education and smoking pack years. The ranges of mean attenuation in traits by these covariates were 9.5 to 15.1% in the discovery set and 4.7 to 20.3% in the replication set (Additional file 3: Tables S7 and S8, respectively). Smoking pack years exhibited the greatest mean attenuation in both cohorts (discovery = 15.1%, replication = 20.3%).

### Epigenetic measures of ageing and disease incidence

**Fig. 2:**
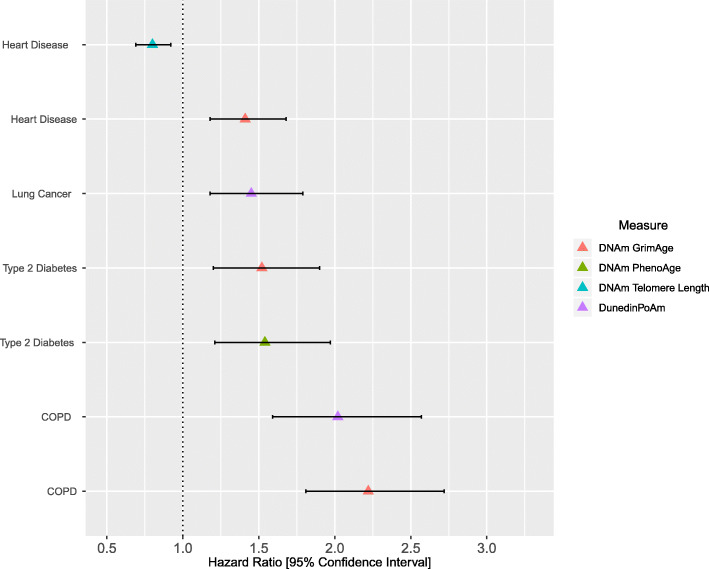
The associations between epigenetic measures of ageing and incidence of common disease states in Generation Scotland. Age-adjusted DNAm GrimAge was associated with the incidence of COPD, type 2 diabetes and ischemic heart disease after 13 years of follow-up. Age-adjusted DNAm PhenoAge associated with the incidence of type 2 diabetes. Age-adjusted measures of DNAm Telomere Length associated with the incidence of ischemic heart disease. Higher DunedinPoAm values, indicating a faster pace of ageing, were associated with the incidence of COPD and lung cancer. Associations represent a one standard deviation increase in the respective epigenetic measure of ageing. Models were adjusted for age, sex, alcohol consumption, body mass index, deprivation, education and smoking. COPD (chronic obstructive pulmonary disease)

For incident disease outcomes, there were 17 Bonferroni-corrected significant associations at *P* < 8.33 × 10^−4^ (*P* < 0.05/60 tests; full output in Additional file 3: Table S9, see also Additional file 4: Fig. S5 and Additional file 6: Note 2). Of these, 7 remained significant in a fully adjusted model at a Bonferroni-corrected significance threshold of 8.33 × 10^−4^ (Additional file 3: Table S10). These relationships are presented herein and in Fig. 2.

A one standard deviation increase in DNAm GrimAge at baseline was associated with the incidence of COPD (HR = 2.22, 95% CI = [1.81, 2.72], *P* = 2.4 × 10^−14^), type 2 diabetes (HR = 1.52, 95% CI = [1.20, 1.90], *P* = 3.1 × 10^−4^) and heart disease (HR = 1.41, 95% CI = [1.18, 1.68], *P* = 1.1 × 10^−4^). Higher values of DunedinPoAm (per SD) associated with the incidence of COPD (HR = 2.02, 95% CI = [1.59, 2.57], *P* = 8.4 × 10^−9^) and lung cancer (HR = 1.45, 95% CI = [1.18, 1.79], *P* = 5.3 × 10^−4^). An accelerated DNAm PhenoAge (per SD) associated with a higher incidence of type 2 diabetes (HR = 1.54, 95% CI = [1.21, 1.97], *P* = 4.5 × 10^−4^). Age-adjusted DNAm Telomere Length (per SD) associated with a lower incidence of heart disease (HR = 0.80, 95% CI = [0.69, 0.92], *P* = 2.5 × 10^−4^).

### Sex-specific analyses of epigenetic measures of ageing and phenotypes in Generation Scotland

As the occurrence of common diseases differs between the sexes, we ran sensitivity analyses using cross-sectional data to determine the correlation between effect sizes for males versus females. In the discovery cohort, continuous phenotypes had a correlation coefficient of 0.93 between sexes whereas categorical disease phenotypes exhibited a correlation coefficient of 0.81 (Additional file 4: Fig. S6). In the replication cohort, there was a correlation of 0.86 and 0.70 between effect sizes for continuous and categorical phenotypes, respectively (Additional file 4: Fig. S7). Excluding diseases ≤ 10 cases (lung and bowel cancer), the largest difference between males and females was for the DunedinPoAm-chronic kidney disease relationship (males: no. of events = 40, OR = 1.23, females: no. of events = 45, OR = 1.72, absolute difference = 0.49). On average, the largest difference between males and females across measures was observed for COPD with males having a higher odds ratio for each measure of ageing (mean difference in effect sizes across measures = 0.25, range = [0.12, 0.37], discovery cohort; Additional file 3: Table S11).

## Discussion

In this study, we examined associations between six major epigenetic measures of ageing and the prevalence and incidence of the leading causes of mortality and disease burden in high-income countries. DNAm GrimAge, a predictor of mortality, associated with the prevalence of COPD and incidence of various disease states, including COPD, type 2 diabetes and cardiovascular disease. It was associated with death due to all-cause mortality and outperformed competitor epigenetic measures of ageing in capturing variability across clinically associated continuous traits. Higher values for DunedinPoAm, which captures faster rates of biological ageing, associated with the incidence of COPD and lung cancer. Higher-than-expected DNAm PhenoAge predicted the incidence of type 2 diabetes in the present study. Age-adjusted measures of DNAm Telomere Length associated with the incidence of ischemic heart disease. Our results replicate previous cross-sectional findings between DNAm PhenoAge and body mass index, diabetes [21] and socioeconomic position (in a basic model) [28]. We also replicated associations between DNAm GrimAge and heart disease [14]. Lastly, we replicated the relationship between Hannum Age and creatinine [31] and between DNAmTLadjAge and smoking pack years [18]. This is also the first external study examining the association between DunedinPoAm and a wide range of health outcomes.

DNAm GrimAge served as a powerful correlate of various phenotypes in our study and has been previously shown to associate with incident heart disease, time-to-cancer and neurological health [14, 22]. DNAm GrimAge is derived from chronological age, sex and methylation-based surrogates of smoking pack years and seven plasma proteins (including DNAm-based estimators of plasminogen activator inhibitor 1, growth differentiation factor 15 and cystatin C). Here, we show that this blood-based epigenetic predictor of mortality risk is associated with poorer performance in lung function tests and predicted incidence of COPD. Compromised lung function has previously been linked to mortality [32, 33]. While it is possible that the associations are mainly driven by the inclusion of smoking pack years, DNAm GrimAge remained associated with COPD and spirometry tests when controlling for self-reported smoking pack years. Similarly, DunedinPoAm associated with time-to-onset of COPD and lung cancer. DunedinPoAm also demonstrated a strong correlation with smoking pack years in our study; however, the associations between DunedinPoAm and incident disease outcomes remained after adjusting for common disease risk factors, including smoking behaviour. In their original study, Belsky et al. identified that the *AHRR* probe cg05575921 was among the 46 CpG sites used to calculate DunedinPoAm. This probe has been strongly associated with smoking behaviour [34–41]. The authors also demonstrated that a version of DunedinPoAm calculated without this probe correlated 0.94 with the DunedinPoAm measure including all probes [20]. DNAm PhenoAge predicted the incidence of type 2 diabetes; however, this may reflect the inclusion of HbA1c in the phenotypic age measure which is used to diagnose diabetes. In our study, an epigenetic predictor of telomere length predicted time-to-onset of ischemic heart disease. A shorter leukocyte telomere length has been shown to associate with heart disease in diverse populations, suggesting that the DNAm Telomere Length predictor may capture key facets of this clinical association [42–44]. Our rich resource of genome-wide DNA methylation and longitudinal health data is the first to show the association of epigenetic measures of ageing with a wide range of common disease states, even after accounting for major confounding influences. These findings have implications for the potential utility of epigenetic measures of ageing in clinical settings.

The majority of our prevalent disease data relied on self-report. Self-report prevalence data have been shown to have a high degree of sensitivity and specificity [45]. Our incident data was obtained using ICD-10 codes from health record linkage. Strikingly, measures of biological ageing showed strong associations with the incidence of common diseases following 13 years of follow-up from the study baseline. These measures performed better at predicting incident rather than prevalent data. However, this may reflect the inclusion of health record-linked versus self-report data and the larger sample size in incidence analyses. Notably, the six epigenetic measures of ageing in our study are correlated with one another among study participants. As well as this, the incidence or prevalence of different disease states, as well as associated continuous traits, may be correlated with one another as they may reflect patterns of poor overall health and disease risk behaviours. Therefore, our application of Bonferroni-corrected significance thresholds is stringent and only captured the most high-confidence associations in our study. These associations were also independent of common disease risk factors and therefore may reflect important associations between age-related physiological changes and risk of disease.

An important limitation is the lack of adjustments for medication use, which may confound associations between epigenetic measures and chronic conditions. Furthermore, studies examining causality between the relationships shown are merited. It is also unclear whether the risk factors examined in this study play a causal role in driving associations between epigenetic measures of ageing and phenotypes, or whether these pleiotropically affect altered DNA methylation and adverse health outcomes. Genetic influences may contribute to differences in DNA methylation and the subsequent estimation of epigenetic age or pace of ageing; therefore, it is possible that our findings may not be generalisable to individuals of non-European ancestry [46, 47].

## Conclusions

In conclusion, using a large cohort with rich health and DNA methylation data, we provide the first comparison of six major epigenetic measures of biological ageing with respect to their associations with leading causes of mortality and disease burden. DNAm GrimAge outperformed the other measures in its associations with disease data and associated clinical traits. This may suggest that predicting mortality, rather than age or homeostatic characteristics, may be more informative for common disease prediction. Thus, proteomic-based methods (as utilised by DNAm GrimAge) using large, physiologically diverse protein sets for predicting ageing and health may be of particular interest in future studies. Our results may help to refine the future use and development of biological age estimators, particularly in studies which aim to comprehensively examine their ability to predict stringent clinically defined outcomes. Our analyses suggest that epigenetic measures of ageing can predict the incidence of common disease states, even after accounting for major confounding risk factors. This may have significant implications for their potential utility in clinical settings to complement gold-standard methods of clinical disease assessment and management.

## Methods

### Generation Scotland

Details of the Generation Scotland (GS) study have been described previously [48, 49]. Briefly, the cohort includes 23,960 individuals, where most individuals (94.2%) have at least one other first-degree family member participating in the study. This encompasses 5573 families with a median family size of 3 (interquartile range = 2–5 members; excluding 1400 singletons without any relatives in the study). For prevalence analyses, the discovery cohort comprised unrelated GS participants with genome-wide methylation data (*n*_discovery_ = 4450). The replication cohort was also derived from GS participants, unrelated to those in the discovery cohort, who had genome-wide DNA methylation measured in a separate batch (*n* = 5087). Within the replication cohort, 2578 participants were also unrelated to one another and these unrelated individuals were considered for cross-sectional analyses (*n*_replication_ = 2578). For incidence analyses, all individuals with available methylation and phenotypic data in GS were considered (*n* = 4450 + 5087 = 9537).

### DNA methylation and calculation of biological ageing measures

DNA methylation levels were measured using the Illumina HumanMethylationEPIC BeadChip Array on blood samples from GS participants. Further details on the processing of DNAm data and the calculation of the six measures of ageing, or pace of ageing, are outlined in Additional file 7; the five clocks (other than DunedinPoAm) were calculated using Horvath’s online age calculator (https://dnamage.genetics.ucla.edu/). Normalised GS methylation data were uploaded as input for the algorithm. Data underwent a further round of normalisation by the age calculator. Briefly, Horvath Age provides an estimate of biological ageing termed “intrinsic epigenetic age acceleration (IEAA)” as it is independent of age-related changes in blood composition. IEAA is derived from regressing Horvath Age onto chronological age. In contrast, Hannum Age provides a measure of ageing referred to as “extrinsic epigenetic age acceleration (EEAA)” as it encompasses age-related changes in blood cell composition. EEAA is derived from regressing a weighted average of Hannum Age and three blood cell types (naive and exhausted cytotoxic T cells, and plasmablasts) onto chronological age. DNAm PhenoAge reflects an individual’s ‘Phenotypic Age’ and, when regressed onto chronological age, provides an index of age acceleration termed ‘AgeAccelPheno’. Similarly, when age-adjusted, DNAm GrimAge is termed ‘AgeAccelGrim’. Lastly, age-adjusted ‘DNAm Telomere Length’ is referred to as ‘DNAmTLadjAge’. DunedinPoAm was calculated using DNAm beta values as input and the *DunedinPoAm38* package in *R* developed by the original study’s authors (https://github.com/danbelsky/DunedinPoAm38 [20]). The five aforementioned epigenetic clocks capture a state of accelerated or decelerated biological ageing reflecting how much ageing has occurred in the individual. However, DunedinPoAm was trained to provide a single time-point, blood-based measurement of the pace of biological ageing in individuals. DunedinPoAm is a DNAm-based proxy of the ‘Pace of Aging’ (PoA) measure. PoA was derived by examining the rate of change in 18 blood-chemistry and organ-system-function biomarkers at three successive time points in participants of Dunedin Study (*n* = 954). The participants were all born in 1972–1973 and were aged 26, 32 and 38 at the time of biomarker measurements. Mixed-effects growth modelling of longitudinal changes in biomarker levels among participants allowed for estimations of the rate of change in biomarker levels for each participant. The sum of random slopes for the biomarker levels (rate of change for each participant) provided a measure of PoA [19]. An elastic net regression model using DNAm data and PoA calculated at age 38 in participants of the Dunedin Study identified 46 CpG sites as informative for predicting PoA, thereby creating a single time-point measure of PoA called DunedinPoAm. DunedinPoAm reflects years of biological ageing per each calendar year. These six measures of biological ageing were input as independent variables in statistical models. Correlations between these predictors are shown in Additional file 4: Fig. S8 for the discovery and replication sets. The correlation structure between these predictors was similar in both sets. DNAmTLadjAge was negatively correlated with the other five indices of ageing (discovery: mean coefficient = − 0.34, range = − 0.12 to − 0.47). This negative correlation was present as shorter telomere lengths typically correspond to an advanced age. The mean correlation coefficient between the remaining five predictors was 0.35 (discovery: range = 0.07 to 0.73).

### Phenotype preparation

For continuous phenotypes, outliers were defined as those values which were beyond 3.5 standard deviations from the mean for a given trait. These outliers were removed prior to analyses. Body mass index was log-transformed. To reduce skewness in the distribution of alcohol consumption and smoking pack years, a log(units +1) or log(pack years +1) transformation was performed. The interval from the start of the Q wave to the end of the T wave on electrocardiogram tests (QT interval) was corrected for heart rate. A general fluid (‘*gf’*) cognitive ability score was derived from principal components analysis of three tests examining different cognitive domains. These domains were processing speed (Wechsler Digit Symbol Substitution Test), verbal declarative memory (Wechsler Logical Memory Test) and verbal fluency (the phonemic verbal fluency test). To derive a general (‘*g’*) cognitive ability score, the principal component analysis was performed on the above three tests and a measure of crystallised intelligence: The Mill Hill Vocabulary test. The first unrotated principal components from these analyses were extracted and labelled as ‘*gf*’ and ‘*g*’, respectively.

For categorical phenotypes, we aimed to examine the ten leading causes of mortality in high-income countries [2]. We also aimed to examine the ten leading causes of disease burden, six of which overlap with the top causes of mortality. This represents fourteen diseases. We had self-report phenotypic information for the prevalence of nine of these diseases (Additional file 1); specifically, we lacked self-report information on lower respiratory diseases and kidney disease (mortality), skin and sense organ diseases (disease burden), and Alzheimer’s disease (AD; present in both the leading causes of mortality and disease burden). We were able to use proxy phenotypes for two of these conditions. We used self-reported maternal history and paternal history as proxies for AD. For kidney disease, we estimated glomerular filtration rate (eGFR) from serum creatinine levels using the chronic kidney disease epidemiology collaboration CKD-EPI equation [50] from which we inferred the prevalence of chronic kidney disease (CKD). Individuals with an eGFR < 60 ml/min/1.73 m^2^ were considered to have CKD. In addition to self-report depression, we also had available information on SCID (Structured Clinical Interview for DSM)-identified depression [51]. Lastly, we separated self-reported back and neck pain into distinct phenotypes for analyses. Together, this resulted in a total of fourteen disease phenotypes for prevalence analyses.

In relation to disease incidence, health record linkage was available for up to 13 years of follow-up since the study baseline (median time-of-onset from baseline = 5.75 years, range = [< 1 month, 13 years]). For each disease state, those individuals who self-reported disease at study baseline were excluded. For cancer, individuals present on the Scottish Cancer Registry (SMR06) were included as cases for incidence analyses. Additionally, for incident cancer analyses, individuals who were recorded on the General Acute Inpatient and Day Case - Scottish Morbidity Records (SMR01) were removed from the control set. For a given condition, individuals who self-reported no disease at study baseline but had prior evidence of diagnosis through health record linkage were removed from analyses. Discovery and replication cohorts were combined to consider all participants for follow-up and to provide a sufficient number of cases for analyses. For incident disease analyses, ICD-10-coded data were retrieved for the following ten conditions: AD, bowel cancer, breast cancer, COPD, depression, type 2 diabetes, dorsalgia (neck and back pain combined), ischemic heart disease, lung cancer and stroke. These reflect the disease states examined in the prevalence analyses with the exception of chronic kidney disease. Furthermore, the two proxies of AD, two measures of depression and separate measures of neck and back pain were replaced by single, clinically defined counterparts in the incidence analyses. Additional file 4: Fig. S9 shows a heatmap for effect sizes from Cox regression models between epigenetic measures of ageing and incident disease outcomes in a fully adjusted model.

### Statistical analyses

Linear regression models were used to examine the association between continuous traits and age-adjusted epigenetic clock measures (reflecting the difference between an individual’s estimated biological age and chronological age) or DunedinPoAm (reflecting the rate of biological ageing). In cross-sectional analyses, logistic regression was used to test the association between categorical disease phenotypes and these epigenetic measures of ageing. In longitudinal analyses, Cox proportional hazards regression models were used to examine whether measures of biological ageing were associated with the incidence of disease. Cox models were also used to examine whether these measures were associated with all-cause mortality in discovery and replication cohorts. There were 182 (4.09%) and 57 (2.25%) deaths in the discovery and replication sets, respectively. The proportional hazards assumption was tested using the *cox.zph()* function in the *survival* package in *R* [52, 53]*.* There was no strong evidence (*P* > 0.05) of assumption violation for the reported significant associations. Phenotypes were scaled to mean zero and unit variance. Continuous or categorical phenotypes were input as dependent variables with measures of biological ageing incorporated as independent variables.

In a basic model, all analyses were adjusted for chronological age and sex. Additional adjustments for height were carried out for measures of lung function. All significant tests from the basic model were then repeated adjusting for additional five covariates, which represent important risk factors for common diseases. These covariates were alcohol intake (units consumed/week), body mass index, educational attainment, deprivation (Scottish Index of Multiple Deprivation) and tobacco smoking pack years.

- *Basic model*: Phenotype ~ Epigenetic Measure + age + sex

b. *Fully adjusted model*: Phenotype ~ Epigenetic Measure + age + sex + alcohol units consumed per week + body mass index + educational attainment + Scottish Index of Multiple Deprivation + smoking pack years

In relation to cross-sectional prevalence data, the discovery analyses consisted of 33 phenotypes which were tested against every epigenetic measure of ageing (all-cause mortality, fourteen disease and eighteen continuous phenotypes; Additional file 3: Table S1). This led to a total of 198 (33 × 6 measures) tests; however, the DNAm GrimAge versus smoking pack years comparison was excluded given the inclusion of a DNAm-based surrogate of pack years in the development of DNAm GrimAge. This led to a Bonferroni-corrected significance threshold of *P* < 0.05/197 tests = 2.54 × 10^-4^. Of these 197 tests, 78 were significant; thus, in the replication cohort, a Bonferroni-corrected significance threshold of *P* < 0.05/78 tests = 6.41 × 10^−4^ was set. In total, 51 associations were significant in both cohorts. The fully adjusted model was then applied to these 51 associations in both the discovery and replication cohorts, holding the same stringent Bonferroni-corrected threshold of *P* < 0.05/78 tests = 6.41 × 10^−4^.

In relation to incidence data, all ten phenotypes were tested against each of the six measures of ageing. In the basic model, this resulted in a Bonferroni-corrected significance threshold of *P* < 0.05/60 tests = 8.33 × 10^−4^. In total, seventeen associations were significant and brought forward to the fully adjusted analysis stage. In the fully adjusted model, the same Bonferroni-corrected significance threshold of *P* < 0.05/60 tests = 8.33 × 10^-4^ was applied.

## Supplementary information

**Additional file 1.** Demographics and Descriptive Statistics for Discovery and Replication Cohorts.

**Additional file 2.** Supplementary Note 1. Significant cross-sectional associations between phenotypes and epigenetic measures of ageing in both discovery and replication cohorts in a basic model adjusting for age and sex.

**Additional file 3.** Supplementary Tables. The associations between epigenetic measures of ageing and disease phenotypes in the discovery cohort (Bonferroni-corrected threshold: P < 2.54 x 10^-4^; significant results are emboldened). (Table S1). The associations between epigenetic measures of ageing and continuous phenotypes in the discovery cohort (Bonferroni-corrected threshold: P < 2.54 x 10^-4^; significant results are emboldened). (Table S2). The associations between epigenetic measures of ageing and all-cause mortality in the discovery cohort (Bonferroni-corrected threshold: P < 2.54 x 10^-4^; significant results are emboldened). (Table S3). Associations between significant phenotypes (identified in the discovery set) and epigenetic measures of ageing in the replication cohort at P < 6.41 x 10^-4^. (Table S4). Discovery Cohort: Associations between phenotypes (significant in basic model) and epigenetic measures of ageing in a fully-adjusted model (Bonferroni threshold: P < 6.41 x 10^-4^). (Table S5). Replication Cohort: Associations between phenotypes (significant in basic model) and epigenetic measures of ageing in a fully-adjusted model (Bonferroni threshold: P < 6.41 x 10^-4^). (Table S6). Covariate-specific analyses of trait-epigenetic age relationship attenuation in the discovery cohort. (Table S7). Covariate-specific analyses of trait-epigenetic age relationship attenuation in the replication cohort. (Table S8). The associations between epigenetic measures of ageing calculated at study baseline and ICD-10-coded incident disease data in Generation Scotland in a basic model adjusted for age and sex. Significant associations that survived a multiple testing correction threshold of 8.33 x 10^-4^ (0.05/60 tests) are emboldened. Nominally significant associations are italicised. (Table S9). The associations between epigenetic measures of ageing measured at study baseline and ICD-10-coded incident disease data in Generation Scotland in a fully-adjusted model adjusted for age, sex and common disease risk factors. Significant associations that survived a multiple testing correction threshold of 8.33 x 10^-4^ (0.05/60 tests) are emboldened. Nominally significant associations are italicised. (Table S10). Sex-specific differences in categorical phenotype-epigenetic age relationships within the discovery cohort. (Table S11).

**Additional file 4.** Significant associations between epigenetic measures of ageing and prevalent disease phenotypes present in both discovery and replication sets in a basic model adjusted for age and sex. (Fig. S1). Significant associations between epigenetic measures of ageing and continuous phenotypes present in both discovery and replication sets in a basic model adjusted for age and sex. (Fig. S2). Associations between epigenetic measures of ageing and all-cause mortality in both discovery (A) and replication (B) sets in a basic model adjusted for age and sex. (Fig. S3). Degree of correlation for continuous variables (A) or categorical variables (B) between discovery and replication cohorts. (Fig. S4). Significant associations between epigenetic measures of ageing and incidence of common disease states in Generation Scotland in a basic model adjusting for age and sex. (Fig. S5). Degree of correlation between males and females in relation to continuous variables (A) or categorical variables (B) in the discovery cohort. (Fig. S6). Degree of correlation between males and females in relation to continuous variables (A) or categorical variables (B) in the replication cohort. (Fig. S7). Correlation structure between different epigenetic measures of biological ageing in discovery (A) and replication (B) sets. (Fig. S8). Heatmap demonstrating the relationship between epigenetic measures of ageing and incident disease outcomes in a fully-adjusted Cox regression model in Generation Scotland. (Fig. S9).

**Additional file 5.** Comparison of epigenetic age measures in terms of their associations with categorical and continuous phenotypes from fully-adjusted models in the replication cohort, stratified by disease type.

**Additional file 6.** Supplementary Note 2. Associations between epigenetic measures of ageing and incidence of ICD-10-coded common diseases in a basic model adjusting for age and sex.

**Additional file 7.** Details of Supplementary Methods.

## Data Availability

According to the terms of consent for GS participants, access to data must be reviewed by the GS Access Committee. Applications should be made to access@generationscotland.org.

## Abbreviations

AD: Alzheimer’s disease
CI: Confidence interval
COPD: Chronic obstructive pulmonary disease
CKD: Chronic kidney disease
DNAm: DNA methylation
DNAm TL: DNAm methylation-based estimator of telomere length
DSM: Diagnostic and Statistical Manual of Mental Disorders
EEAA: Extrinsic epigenetic age acceleration
eGFR: Estimated glomerular filtration rate
FEF: Forced expiratory flow
FEV: Forced expiratory volume
GS: Generation Scotland
GS:SFHS: Generation Scotland: Scottish Family Health Study
HR: Hazard ratio
ICD-10: International Classification of Diseases, 10th Revision
IEAA: Intrinsic epigenetic age acceleration
OR: Odds ratio
SCID: Structured Clinical Interview for Diagnostic and Statistical Manual of Mental Disorders
SD: Standard deviation
SIMD: Scottish index of multiple deprivation
SMR: Scottish morbidity records
TL: Telomere length
