# Supplementary material for: Epigenetic measures of ageing predict the prevalence and incidence of leading causes of death and disease burden
Source: Clin Epigenetics. 2020 Jul 31;12:115. doi: 10.1186/s13148-020-00905-6 (PMC7394682; doi:10.1186/s13148-020-00905-6)
Supplement: Supplementary file 1 — Additional file 1. Demographics and Descriptive Statistics for Discovery and Replication Cohorts. [file 13148_2020_905_MOESM1_ESM.pdf]

Additional file 1. Demographics and Descriptive Statistics for Discovery and Replication Cohorts.

	**Discovery**			**Replication**		
Variable	*n*	*Mean*	*SD*	*n*	*Mean*	*SD*
Age (years)	4450	51.40	13.20	2578	50.00	12.50
	n	*Females/Males*	*%Female*	*n*	*Females/Males*	*%Female*
Sex	4450	2506/1944	56.31%	2578	1583/995	61.40%
Epigenetic Age Measures	*n*	*Mean*	*SD*	*n*	*Mean*	*SD*
DNAm GrimAge (years)	4450	48.82	10.89	2578	60.47	10.63
DNAm PhenoAge (years)	4450	43.73	11.45	2578	44.98	9.75
Horvath Age (EEAA; years)	4450	60.13	9.83	2578	54.75	9.35
Hannum Age (IEAA; years)	4450	47.38	9.64	2578	46.97	9.13
DNAm Telomere Length (TL; kbp)	4450	7.42	0.26	2578	7.31	0.26
DunedinPoAm (years)	4450	1.05	0.07	2578	1.03	0.09
Continuous Phenotypes	*n*	*Mean*	*SD*	*n*	*Mean*	*SD*
Average Heart Rate (beats/min)	4444	69.20	11.40	2572	69.70	11.10
Body Mass Index (kg/m^2)	4423	26.80	4.96	2567	27.20	5.35
Creatinine (µmol/L)	4427	71.20	14.60	2566	70.10	14.50
Diastolic Pressure (mmHg)	4447	80.80	10.50	2573	80.10	9.93
Forced Expiratory Flow (L/s)	3750	2.99	1.23	2191	2.99	1.21
Forced Expiratory Volume (L)	3776	3.02	0.84	2191	3.00	0.82
Forced Vital Capacity (L)	3775	3.97	0.99	2197	3.93	0.97
General Factor of Fluid Intelligence	4324	-0.06	0.99	2529	0.02	0.99
General Factor of Intelligence	4291	0.03	1.01	2504	0.08	0.99
Glucose (mmol/L)	4297	4.78	0.58	2511	4.72	0.60
HDL Cholesterol (mmol/L)	4396	1.46	0.40	2546	1.48	0.41
Neuroticism	4426	3.60	3.09	2565	4.24	3.31
Pack Years	4380	7.54	14.30	2522	8.65	15.50
QT Interval (corrected for heart rate; milliseconds)	4374	7.70	24.20	2542	0.17	23.60
Scottish Index of Multiple Deprivation (rank 1 : 6,505 = most to least deprived)	4236	3980.00	1840.00	2457	3900.00	1890.00
Systolic Pressure (mmHg)	4447	134.00	18.40	2573	132.00	17.10
Total Cholesterol (mmol/L)	4409	5.21	1.05	2551	5.19	1.06
Waist : Hip Ratio	4383	0.87	0.09	2535	0.87	0.10

Ordinal Variables	*n*	*Median*	*IQR*	*n*	*Median*	*IQR*
Educational Attainment (years)	4303	4	3	2434	4	3
Self-Report Diseases	*n*	*No. of Cases*	*% Cases*	*n*	*No. of Cases*	*%Cases*
Alzheimer's Disease Maternal	4450	228	5.12%	2531	128	5.06%
Alzheimer's Disease Paternal	4450	134	3.01%	2531	87	3.44%
Back Pain	1693	480	28.35%	1475	295	20.00%
Bowel Cancer	4450	20	0.45%	2531	11	0.43%
Breast Cancer	4450	63	1.42%	2531	40	1.58%
Chronic Kidney Disease	4427	85	1.92%	2563	40	1.56%
COPD	4450	48	1.08%	2531	32	1.26%
Depression	4450	371	8.34%	2531	414	16.36%
Diabetes	4450	147	3.30%	2531	89	3.52%
Heart Disease	4450	196	4.40%	2531	95	3.75%
Lung Cancer	4450	5	0.11%	2531	4	0.16%
Neck Pain	1693	431	25.46%	1475	257	17.42%
SCID Depression	4450	825	18.54%	2578	984	38.17%
Stroke	4450	59	1.33%	2531	41	1.62%
