# Supplementary material for: Epigenetic measures of ageing predict the prevalence and incidence of leading causes of death and disease burden
Source: Clin Epigenetics. 2020 Jul 31;12:115. doi: 10.1186/s13148-020-00905-6 (PMC7394682; doi:10.1186/s13148-020-00905-6)
Supplement: Supplementary file 2 — Additional file 2. Supplementary Note 1. Significant cross-sectional associations between phenotypes and epigenetic measures of ageing in both discovery and replication cohorts in a basic model adjusting for age and sex. [file 13148_2020_905_MOESM2_ESM.docx]

**Additional file 2 - Supplementary Note 1. Significant cross-sectional associations between phenotypes and epigenetic measures of ageing in both discovery and replication cohorts in a basic model adjusting for age and sex.**

*Cardiovascular Diseases*

AgeAccelGrim alone was associated with heart disease (Odds Ratio (OR) per SD = 1.71, P = 1.0 x 10^-7^) but not stroke following replication.

In relation to continuous traits, AgeAccelGrim, DunedinPoAm, AgeAccelPheno and EEAA were positively associated with average heart rate (β = [0.08, 0.18], P = [1.1 x 10^-4^, 1.1 x 10^-19^]), with AgeAccelGrim showing the strongest association in the replication set. AgeAccelPheno and IEAA were positively associated with body mass index (β = [0.07, 0.11], P = [5.5 x 10^-4^, 2.7 x 10^-8^]). AgeAccelPheno was positively associated with higher diastolic blood pressure (β = 0.10, P = 2.4 x 10^-7^). AgeAccelGrim and DunedinPoAm were negatively associated with high-density lipoprotein cholesterol (β = -0.11 and -0.10, P = 1.9 x 10^-8^ and 2.4 x 10^-7^, respectively). AgeAccelGrim and AgeAccelPheno were positively associated with waist-to-hip ratio (β = 0.15 and 0.10, P = 1.1 x 10^-16^ and 1.6 x 10^-8^, respectively). DNAmTLadjAge was negatively associated with average heart rate (β = -0.08, P = 1.7 x 10^-4^) and waist-to-hip ratio (β = -0.08, P = 7.6 x 10^-6^).

*Neurological and Psychiatric Diseases*

AgeAccelGrim was associated with both self-reported depression (OR = 1.26, P = 8.7 x 10^-5^) and SCID Depression (OR = 1.18, P = 6.6 x 10^-5^). None of the measures were associated with either maternal or paternal history of Alzheimer’s disease.

AgeAccelGrim and DunedinPoAm were both negatively associated with general cognitive ability (β = -0.14 and -0.13, P = 1.2 x 10^-11^ and 1.5 x 10^-11^, respectively) and a general factor of fluid intelligence (β = -0.14 and -0.11, P = 1.5 x 10^-12^ and 6.1 x 10^-9^, respectively). AgeAccelGrim and DunedinPoAm were also associated with neuroticism (β = 0.11 and 0.08, P = 4.8 x 10^-8^ and 7.8 x 10^-5^, respectively). DunedinPoAm, AgeAccelGrim and AgeAccelPheno were negatively associated with the Scottish Index of Multiple Deprivation (SIMD; lower indices correspond to more deprivation) (β = [-0.12, -0.27], P = [1.0 x 10^-9^, 1.7 x 10^-38^]). DNAmTLadjAge was positively associated with SIMD (β = 0.15, P = 4.6 x 10^-13^).

*Pulmonary Diseases*

DunedinPoAm and AgeAccelGrim were associated with COPD (OR = 3.42 and 3.49, P = 3.6 x 10^-11^ and 1.4 x 10^-13^, respectively). No measure of epigenetic ageing was associated with lung cancer (no. of events = 5).

AgeAccelGrim, AgeAccelPheno and DunedinPoAm were negatively associated with forced expiratory flow (β = [-0.07,-0.08], P = [1.7 x 10^-5^, 2.6 x 10^-23^]) and forced expiratory volume (β = [-0.06, -0.15], P = [4.3 x 10^-5^, 5.7 x 10^-24^]). AgeAccelGrim and DunedinPoAm were also negatively associated with forced vital capacity (β = -0.08 and -0.05, P = 6.5 x 10^-8^ and 2.7 x 10^-4^, respectively). DunedinPoAm was strongly associated with smoking pack years, representing the strongest association overall (β = 0.38, P = 4.1 x 10^-91^). AgeAccelPheno was also positively associated with smoking pack years (β = 0.17, P = 1.4 x 10^-17^). DNAmTLadjAge was positively associated with forced expiratory flow (β = 0.08, P = 1.4 x 10^-5^), forced expiratory volume (β = 0.07, P = 5.4 x 10^-6^) and negatively associated with pack years (β = -0.20, P = 2.5 x 10^-23^).

*Diabetes Mellitus and Kidney Disease*

AgeAccelGrim and AgeAccelPheno were associated with diabetes (OR: 1.47 and 1.48, P = 1.1 x 10^-4^ and 8.7 x 10^-5^, respectively).

AgeAccelGrim and EEAA were positively associated with creatinine (β = 0.08 and 0.14, P = 4.6 x 10^-5^ and 5.0 x 10^-13^, respectively). DNAmTLadjAge was negatively associated with creatinine (β = -0.11, P = 2.0 x 10^-8^).

*Cancer*

None of the ageing measures were associated with either bowel or breast cancer following multiple testing correction.

*Neck and Back Pain*

DunedinPoAm and AgeAccelGrim were associated with back pain (OR = 1.29 and 1.34, P = 1.9 x 10^-4^, 5.4 x 10^-6^, respectively). No measures were associated with neck pain.

Table 1. Significant relationships between phenotypes and epigenetic measures of ageing present in both discovery and replication cohorts in a basic model. Those phenotypes which remained significant in both cohorts in a subsequent fully-adjusted model are emboldened.

|  |  | Discovery Cohort | | | Replication Cohort | | |
| --- | --- | --- | --- | --- | --- | --- | --- |
| *Categorical Phenotypes* | | | | | | | |
| Measure | Variable | n event | OR | P | n event | OR | P |
| GrimAge | SCID Depression | 825 | 1.45 | 1.8 x 10^-22^ | 984 | 1.18 | 6.6 x 10^-05^ |
| GrimAge | Depression | 371 | 1.62 | 2.5 x 10^-22^ | 414 | 1.26 | 8.7 x 10^-05^ |
| **GrimAge** | **COPD** | **48** | **2.37** | **1.4 x 10^-12^** | **32** | **3.49** | **1.4 x 10^-13^** |
| GrimAge | Heart Disease | 196 | 1.54 | 5.2 x 10^-10^ | 95 | 1.71 | 1.0 x 10^-07^ |
| GrimAge | Diabetes | 147 | 1.58 | 1.4 x 10^-09^ | 89 | 1.47 | 1.1 x 10^-04^ |
| PhenoAge | Diabetes | 147 | 1.55 | 2.3 x 10^-08^ | 89 | 1.48 | 8.7 x 10^-05^ |
| DunedinPoAm | COPD | 48 | 1.89 | 3.4 x 10^-7^ | 32 | 3.42 | 3.6 x 10^-11^ |
| DunedinPoAm | Back Pain | 480 | 1.23 | 4.2 x 10^-5^ | 293 | 1.29 | 1.9 x 10^-04^ |
| GrimAge | Back Pain | 480 | 1.26 | 1.7 x 10^-05^ | 293 | 1.34 | 5.4 x 10^-06^ |
| *Continuous Phenotypes* | | | | | | | |
| Measure | Variable | n | β | P | n | β | P |
| **DunedinPoAm** | **Pack Years** | **4380** | **0.46** | **5.0 x 10^-234^** | **2522** | **0.38** | **4.1 x 10^-91^** |
| **DunedinPoAm** | **SIMD** | **4236** | **-0.24** | **8.7 x 10^-57^** | **2457** | **-0.24** | **9.1 x 10^-34^** |
| **GrimAge** | **SIMD** | **4236** | **-0.28** | **3.3 x 10^-72^** | **2457** | **-0.27** | **1.7 x 10^-38^** |
| **HannumAge** | **Creatinine** | **4427** | **0.24** | **2.8 x 10^-62^** | **0.14** | **0.02** | **5.0 x 10^-13^** |
| **DunedinPoAm** | **Average Heart Rate** | **4444** | **0.21** | **4.0 x 10^-48^** | **2572** | **0.13** | **2.1 x 10^-11^** |
| **GrimAge** | **FEV** | **3750** | **-0.16** | **1.1 x 10^-42^** | **2191** | **-0.15** | **5.7 x 10^-24^** |
| GrimAge | g | 4291 | -0.22 | 6.1 x 10^-42^ | 2504 | -0.14 | 1.2 x 10^-11^ |
| **GrimAge** | **Average Heart Rate** | **4444** | **0.20** | **1.6 x 10^-37^** | **2572** | **0.18** | **1.1 x 10^-19^** |
| GrimAge | gf | 4324 | -0.19 | 5.9 x 10^-37^ | 2529 | -0.14 | 1.5 x 10^-12^ |
| DunedinPoAm | g | 4291 | -0.19 | 2.1 x 10^-36^ | 2504 | -0.13 | 1.5 x 10^-11^ |
| GrimAge | Waist:Hip Ratio | 4383 | 0.15 | 6.5 x 10^-33^ | 2535 | 0.15 | 1.1 x 10^-16^ |
| DunedinPoAm | gf | 4324 | -0.17 | 8.4 x 10^-31^ | 2529 | -0.11 | 6.1 x 10^-9^ |
| **GrimAge** | **FEF** | **3750** | **-0.16** | **1.8 x 10^-29^** | **2185** | **-0.18** | **2.6 x 10^-23^** |
| DunedinPoAm | FEV | 3776 | -0.12 | 7.8 x 10^-29^ | 2191 | -0.10 | 1.9 x 10^-12^ |
| **DNAmTL** | **Pack Years** | **4380** | **-0.17** | **3.0 x 10^-28^** | **2522** | **-0.20** | **2.5 x 10^-23^** |
| **PhenoAge** | **Body Mass Index** | **4423** | **0.16** | **1.4 x 10^-26^** | **2567** | **0.11** | **2.7 x 10^-08^** |
| **PhenoAge** | **Pack Years** | **4380** | **0.15** | **2.4 x 10^-24^** | **2522** | **0.17** | **1.4 x 10^-17^** |
| DunedinPoAm | HDL Cholesterol | 4396 | -0.14 | 2.8 x 10^-23^ | 2543 | -0.10 | 2.4 x 10^-7^ |
| GrimAge | FVC | 3775 | -0.12 | 1.1 x 10^-22^ | 2191 | -0.08 | 6.5 x 10^-08^ |
| DunedinPoAm | FEF | 3750 | -0.13 | 4.3 x 10^-21^ | 2185 | -0.13 | 8.3 x 10^-14^ |
| PhenoAge | Waist:Hip Ratio | 4383 | 0.11 | 1.3 x 10^-20^ | 2535 | 0.10 | 1.6 x 10^-08^ |
| GrimAge | HDL Cholesterol | 4396 | -0.14 | 1.4 x 10^-20^ | 2543 | -0.11 | 1.9 x 10^-08^ |
| **GrimAge** | **Creatinine** | **4427** | **0.14** | **2.5 x 10^-20^** | **2563** | **0.08** | **4.6 x 10^-05^** |
| **PhenoAge** | **Average Heart Rate** | **4444** | **0.13** | **3.3 x 10^-19^** | **2572** | **0.14** | **1.6 x 10^-13^** |
| DNAmTL | SIMD | 4236 | 0.13 | 5.5 x 10^-17^ | 2457 | 0.15 | 4.6 x 10^-13^ |
| DunedinPoAm | FVC | 3775 | -0.13 | 4.3 x 10^-21^ | 2191 | -0.05 | 2.7 x 10^-04^ |
| DNAmTL | Creatinine | 4427 | -0.11 | 3.3 x 10^-14^ | 2563 | -0.11 | 2.0 x 10^-08^ |
| PhenoAge | SIMD | 4236 | -0.11 | 7.7 x 10^-14^ | 2457 | -0.12 | 1.0 x 10^-09^ |
| HannumAge | Average Heart Rate | 4444 | 0.11 | 9.2 x 10^-13^ | 2572 | 0.08 | 1.1 x 10^-04^ |
| DunedinPoAm | Body Mass Index | 4432 | 0.10 | 1.8 x 10^-11^ | 22567 | 0.07 | 5.5 x 10^-04^ |
| PhenoAge | FEV | 3776 | -0.07 | 1.5 x 10^-09^ | 2191 | -0.06 | 4.3 x 10^-05^ |
| DNAmTL | Average Heart Rate | 4444 | -0.09 | 4.4 x 10^-09^ | 2572 | -0.08 | 1.7 x 10^-04^ |
| GrimAge | Neuroticism | 4426 | 0.08 | 5.7 x 10^-08^ | 2565 | 0.11 | 4.8 x 10^-08^ |
| DunedinPoAm | Neuroticism | 4426 | 0.07 | 4.6 x 10^-07^ | 2565 | 0.08 | 7.8 x 10^-05^ |
| DNAmTL | FEV | 3776 | 0.05 | 3.9 x 10^-06^ | 2191 | 0.06 | 5.4 x 10^-06^ |
| PhenoAge | Diastolic Pressure | 4447 | 0.06 | 4.3 x 10^-06^ | 2573 | 0.10 | 2.4 x 10^-07^ |
| DNAmTL | Waist:Hip Ratio | 4383 | -0.06 | 6.9 x 10^-06^ | 2535 | -0.08 | 7.6 x 10^-07^ |
| HorvathAge | Body Mass Index | 4423 | 0.06 | 4.2 x 10^-05^ | 2567 | 0.09 | 1.2 x 10^-05^ |
| PhenoAge | FEF | 3750 | -0.06 | 4.6 x 10^-05^ | 2185 | -0.07 | 1.7 x 10^-05^ |
| DNAmTL | FEF | 3750 | 0.06 | 5.0 x 10^-05^ | 2185 | 0.07 | 1.4 x 10^-04^ |
| *Mortality Analysis* | | | | | | | |
| Measure | Variable | n event | HR | P | n events | HR | P |
| **GrimAge** | **All-Cause Mortality** | **182** | **1.87** | **<2.0 x 10^-16^** | **57** | **1.7** | **6.5 x 10^-05^** |
| DunedinPoAm | All-Cause Mortality | 182 | 1.7 | 5.4 x 10^-15^ | 57 | 1.69 | 8.1 x 10^-05^ |

COPD (chronic obstructive pulmonary disease), FEF (forced expiratory flow), FEV (forced expiratory volume), FVC (forced vital capacity), g (general factor of cognitive ability), gf (general factor of fluid intelligence), HDL (high-density lipoprotein), HR (hazard ratio), OR (odds ratio), SCID (Structured Clinical Interview for DSM), SIMD (Scottish Index of Multiple Deprivation).
