# Supplementary material for: Epigenetic measures of ageing predict the prevalence and incidence of leading causes of death and disease burden
Source: Clin Epigenetics. 2020 Jul 31;12:115. doi: 10.1186/s13148-020-00905-6 (PMC7394682; doi:10.1186/s13148-020-00905-6)
Supplement: Supplementary file 4 — Additional file 4. Significant associations between epigenetic measures of ageing and prevalent disease phenotypes present in both discovery and replication sets in a basic model adjusted for age and sex. (Fig. S1). Significant associations between epigenetic measures of ageing and continuous phenotypes present in both discovery and replication sets in a basic model adjusted for age and sex. (Fig. S2). Associations between epigenetic measures of ageing and all-cause mortality in both discovery (A) and replication (B) sets in a basic model adjusted for age and sex. (Fig. S3). Degree of correlation for continuous variables (A) or categorical variables (B) between discovery and replication cohorts. (Fig. S4). Significant associations between epigenetic measures of ageing and incidence of common disease states in Generation Scotland in a basic model adjusting for age and sex. (Fig. S5). Degree of correlation between males and females in relation to continuous variables (A) or categorical variables (B) in the discovery cohort. (Fig. S6). Degree of correlation between males and females in relation to continuous variables (A) or categorical variables (B) in the replication cohort. (Fig. S7). Correlation structure between different epigenetic measures of biological ageing in discovery (A) and replication (B) sets. (Fig. S8). Heatmap demonstrating the relationship between epigenetic measures of ageing and incident disease outcomes in a fully-adjusted Cox regression model in Generation Scotland. (Fig. S9). [file 13148_2020_905_MOESM4_ESM.docx]

**Additional file 4 – Supplementary Figures**

**Fig. S1. Significant associations between epigenetic measures of ageing and prevalent disease phenotypes present in both discovery and replication sets in a basic model adjusted for age and sex.** A higher-than-expected DNAm GrimAge (higher AgeAccelGrim) was positively associated with SCID depression and self-reported measures of depression, back pain, diabetes, heart disease and COPD. A higher-than-expected DNAm PhenoAge (higher AgeAccelPheno) was positively associated with diabetes. Higher values of DunedinPoAm (a faster pace of ageing) were associated with back pain and COPD. COPD (Chronic Obstructive Pulmonary Disease), SCID (Structured Clinical Interview for DSM). Replication set statistics are presented.

**Fig. S2. Significant associations between epigenetic measures of ageing and continuous phenotypes present in both discovery and replication sets in a basic model adjusted for age and sex.** AgeAccelGrim (DNAm GrimAge) was negatively associated with SIMD, forced expiratory flow, forced expiratory volume, a general factor of fluid intelligence (gf), a general factor of cognitive ability (g), HDL cholesterol and forced vital capacity. AgeAccelGrim was positively associated with creatinine, neuroticism, waist-to-hip ratio and average heart rate. AgeAccelPheno (DNAm PhenoAge) was negatively associated with SIMD, forced expiratory flow and forced expiratory volume. AgeAccelPheno was positively associated with waist-to-hip ratio, diastolic pressure, body mass index, average heart rate and smoking pack years. DNAmTLadjAge (DNAm Telomere Length) was negatively associated with smoking pack years, creatinine, waist-to-hip ratio and average heart rate. DNAmTLadjAge was positively associated with forced expiratory volume, forced expiratory flow and SIMD. DunedinPoAm was negatively associated with SIMD, forced expiratory flow, g, gf, forced expiratory volume, HDL cholesterol and forced vital capacity. DunedinPoAm was positively associated with body mass index, neuroticism, average heart rate and smoking pack years. EEAA (Hannum Age) was positively associated with average heart rate and creatinine. IEAA (Horvath Age) was positively associated with body mass index. HDL (High-density Lipoprotein), SIMD (Scottish Index of Multiple Deprivation). Replication set statistics are presented.

**Fig. S3. Associations between epigenetic measures of ageing and all-cause mortality in both discovery (A) and replication (B) sets in a basic model adjusted for age and sex.** In the discovery (A) and replication (B) cohorts, there were 182 (4.09%) and 57 (2.25%) deaths, respectively. Following correction for multiple testing, AgeAccelGrim (DNAm GrimAge) and DunedinPoAm were significantly associated with all-cause mortality in the discovery (n = 4450) and replication (n = 2578) cohorts.

**Fig. S4. Degree of correlation for continuous variables (A) or categorical variables (B) between discovery and replication cohorts.** Lung and bowel cancer were excluded due to a low number of cases < 10.

**Fig. S5. Significant associations between epigenetic measures of ageing and incidence of common disease states in Generation Scotland in a basic model adjusting for age and sex.** Age-adjusted DNAm GrimAge was associated with incidence of COPD, diabetes, lung cancer, ischemic heart disease and stroke after thirteen years of follow-up. Age-adjusted DNAm PhenoAge associated with incidence of diabetes, COPD and ischemic heart disease. Age-adjusted measures of DNAm Telomere Length associated with incidence of heart disease, diabetes, stroke and COPD. Higher values of DunedinPoAm were associated ischemic heart disease, lung cancer, stroke, diabetes and COPD. COPD (Chronic Obstructive Pulmonary Disease).

**Fig. S6. Degree of correlation between males and females in relation to continuous variables (A) or categorical variables (B) in the discovery cohort.** Lung and bowel cancer were excluded due to a low number of cases < 10. Breast cancer was excluded as only female cases were present in Generation Scotland.

**Fig. S7. Degree of correlation between males and females in relation to continuous variables (A) or categorical variables (B) in the replication cohort.** Lung and bowel cancer were excluded due to a low number of cases < 10. Breast cancer was excluded as only female cases were present in Generation Scotland.

**Fig. S8. Correlation structure between different epigenetic measures of biological ageing in discovery (A) and replication (B) sets.** All measures of biological ageing were positively correlated with one another, with the exception of age-adjusted DNAm Telomere Length (DNAmTLAdjAge). AgeAccelGrim and AgeAccelPheno refer to age-adjusted DNAm GrimAge and DNAm PhenoAge, respectively. EEAA (Hannum Age), IEAA (Horvath Age).


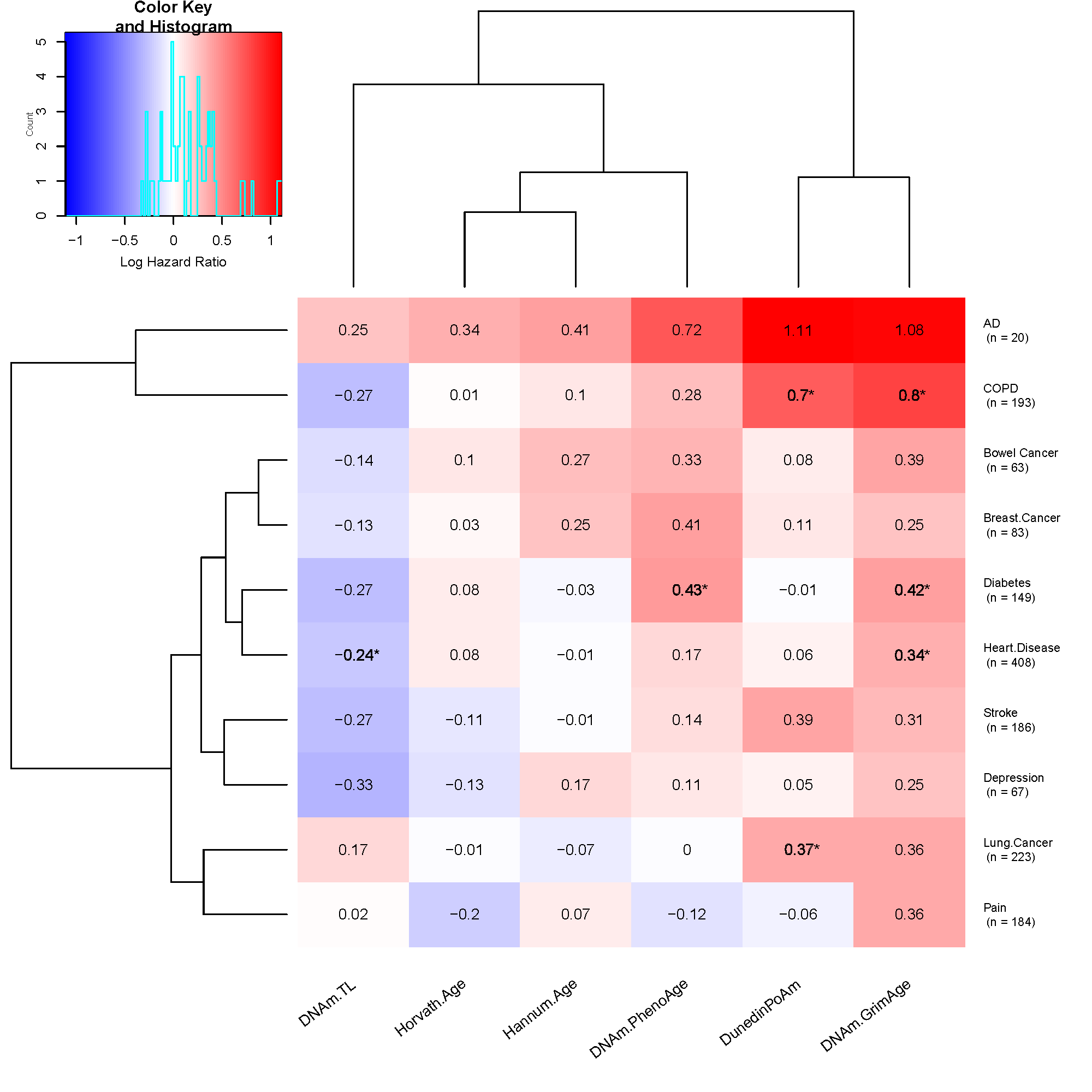


**Fig S9. Heatmap demonstrating the relationship between epigenetic measures of ageing and incident disease outcomes in a fully-adjusted Cox regression model in Generation Scotland.** The heatmap shows log Hazard Ratios derived from Cox regression models examining the relationship between six major epigenetic measures of ageing and incident disease outcomes after 13 years of follow-up. These models were adjusted for age, sex and five common disease risk factors, reflecting output from the fully-adjusted analyses. A demarcation is set at 0 to distinguish between associations whereby a higher value for an epigenetic measure of ageing indicates a protective effect (log Hazard Ratio < 0 or Hazard Ratio < 1) or a risk effect (log Hazard Ratio > 0 or Hazard Ratio > 1). Importantly, these Hazard Ratios do not reflect the significance of the associations as information relating to number of cases (shown under disease names) or standard errors are not captured by the heatmap. Associations which were significant at a Bonferroni-corrected significance threshold are emboldened and marked with an asterisk. AD (Alzheimer’s Disease), COPD (Chronic Obstructive Pulmonary Disease), TL (Telomere Length).
