# Supplementary material for: Epigenetic measures of ageing predict the prevalence and incidence of leading causes of death and disease burden
Source: Clin Epigenetics. 2020 Jul 31;12:115. doi: 10.1186/s13148-020-00905-6 (PMC7394682; doi:10.1186/s13148-020-00905-6)
Supplement: Supplementary file 5 — Additional file 5. Comparison of epigenetic age measures in terms of their associations with categorical and continuous phenotypes from fully-adjusted models in the replication cohort, stratified by disease type. [file 13148_2020_905_MOESM5_ESM.pdf]

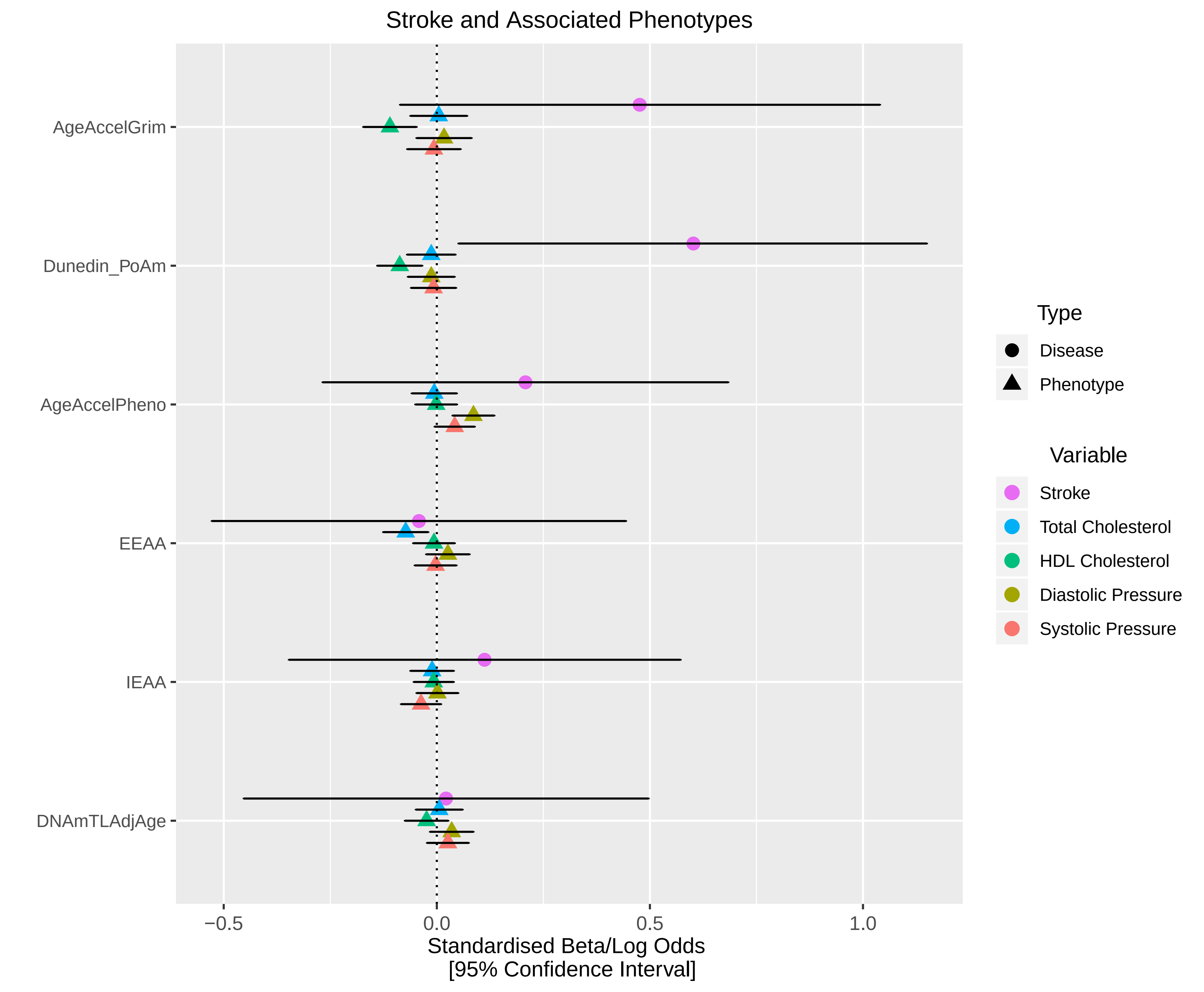
Stroke and Associated Phenotypes
AgeAccelGrim
Dunedin_PoAm
AgeAccelPheno
EEAA
IEAA
DNAmTLAdjAge
−0.5
0.0
0.5
1.0
Standardised Beta/Log Odds
[95% Confidence Interval]
Type
Disease
Phenotype
Variable
Stroke
Total Cholesterol
HDL Cholesterol
Diastolic Pressure
Systolic Pressure

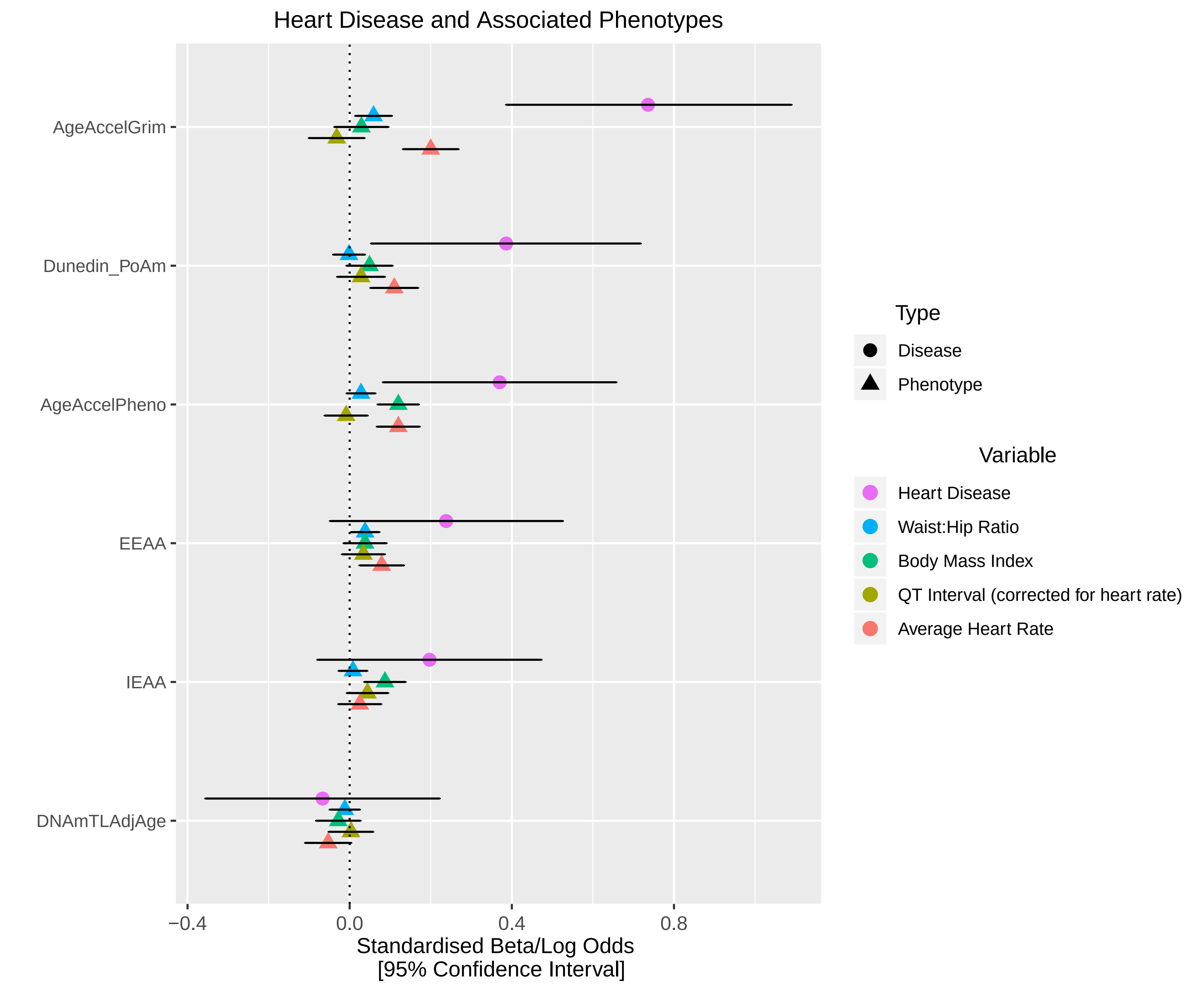

Heart Disease and Associated Phenotypes
AgeAccelGrim
Dunedin_PoAm
AgeAccelPheno
EEAA
IEAA
DNAmTLAdjAge
−0.4
0.0
0.4
0.8
Standardised Beta/Log Odds
[95% Confidence Interval]
Type
Disease
Phenotype
Variable
Heart Disease
Waist:Hip Ratio
Body Mass Index
QT Interval (corrected for heart rate)
Average Heart Rate

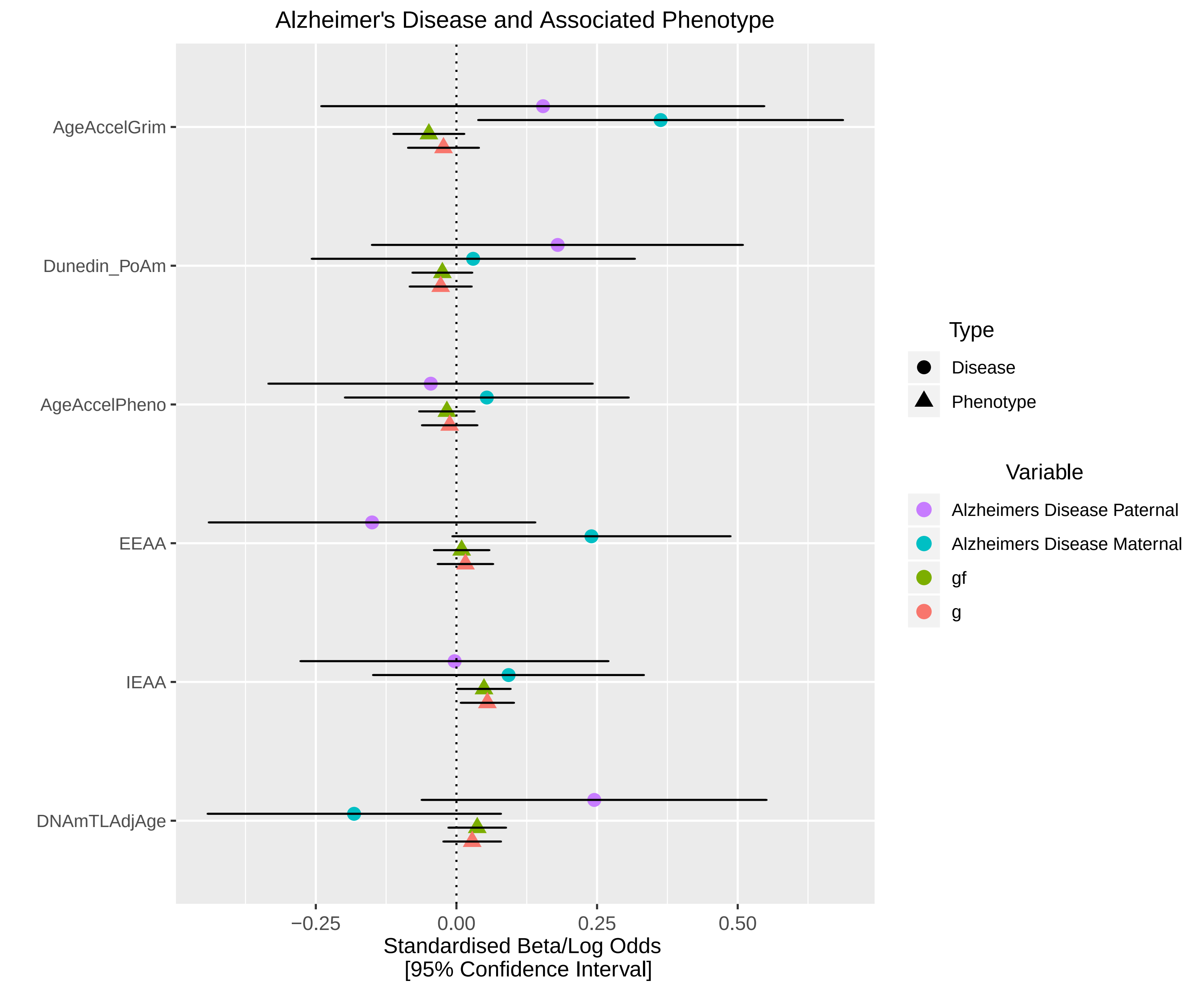

Alzheimer's Disease and Associated Phenotype
AgeAccelGrim
Dunedin_PoAm
AgeAccelPheno
EEAA
IEAA
DNAmTLAdjAge
−0.25
0.00
0.25
0.50
Standardised Beta/Log Odds
[95% Confidence Interval]
Type
Disease
Phenotype
Variable
Alzheimers Disease Paternal
Alzheimers Disease Maternal
gf
g

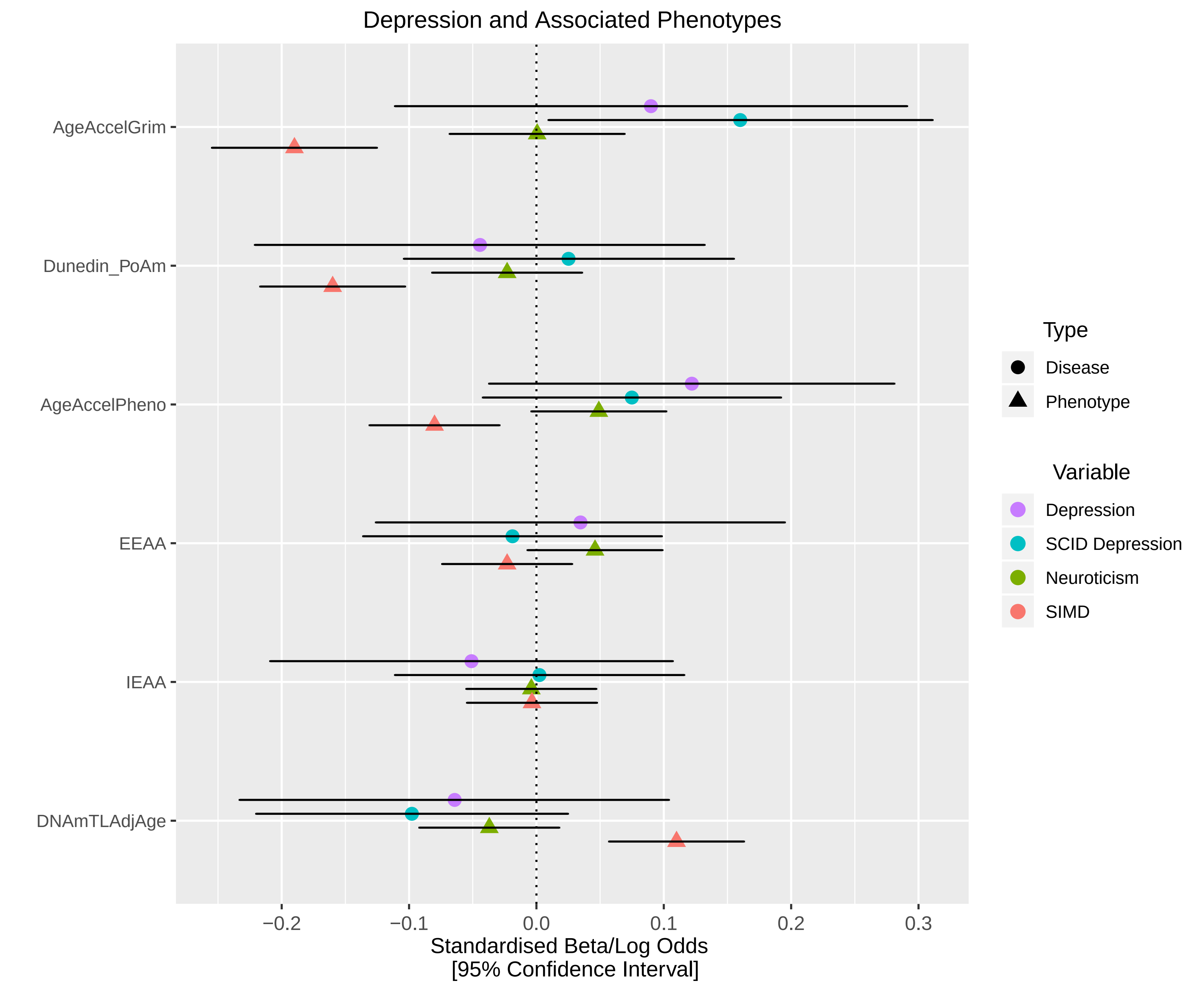

Depression and Associated Phenotypes
AgeAccelGrim
Dunedin_PoAm
AgeAccelPheno
EEAA
IEAA
DNAmTLAdjAge
−0.2
−0.1
0.0
0.1
0.2
0.3
Standardised Beta/Log Odds
[95% Confidence Interval]
Type
Disease
Phenotype
Variable
Depression
SCID Depression
Neuroticism
SIMD

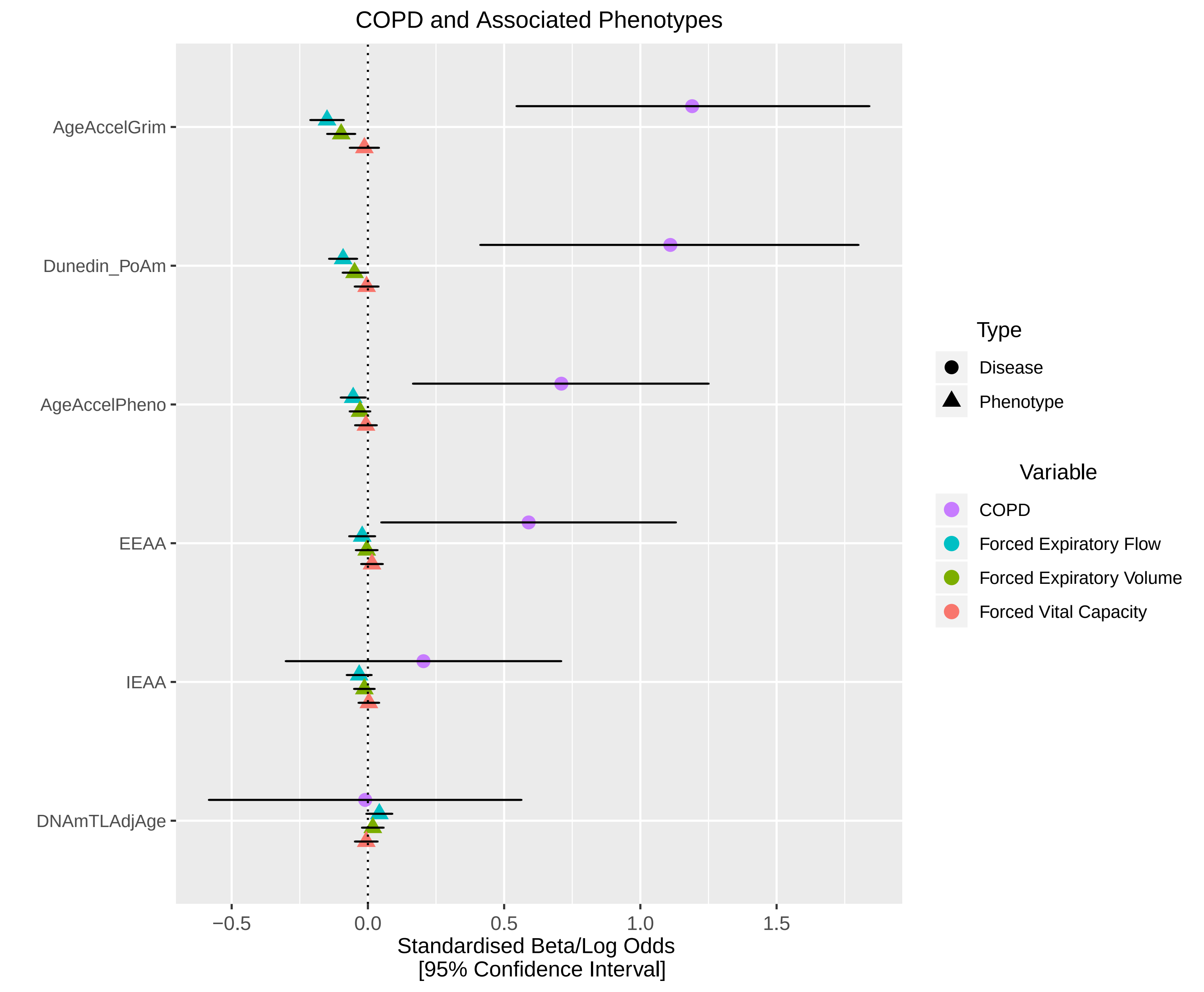
COPD and Associated Phenotypes
AgeAccelGrim
Dunedin_PoAm
AgeAccelPheno
EEAA
IEAA
DNAmTLAdjAge
−0.5
0.0
0.5
1.0
1.5
Standardised Beta/Log Odds
[95% Confidence Interval]
Type
Disease
Phenotype
Variable
COPD
Forced Expiratory Flow
Forced Expiratory Volume
Forced Vital Capacity

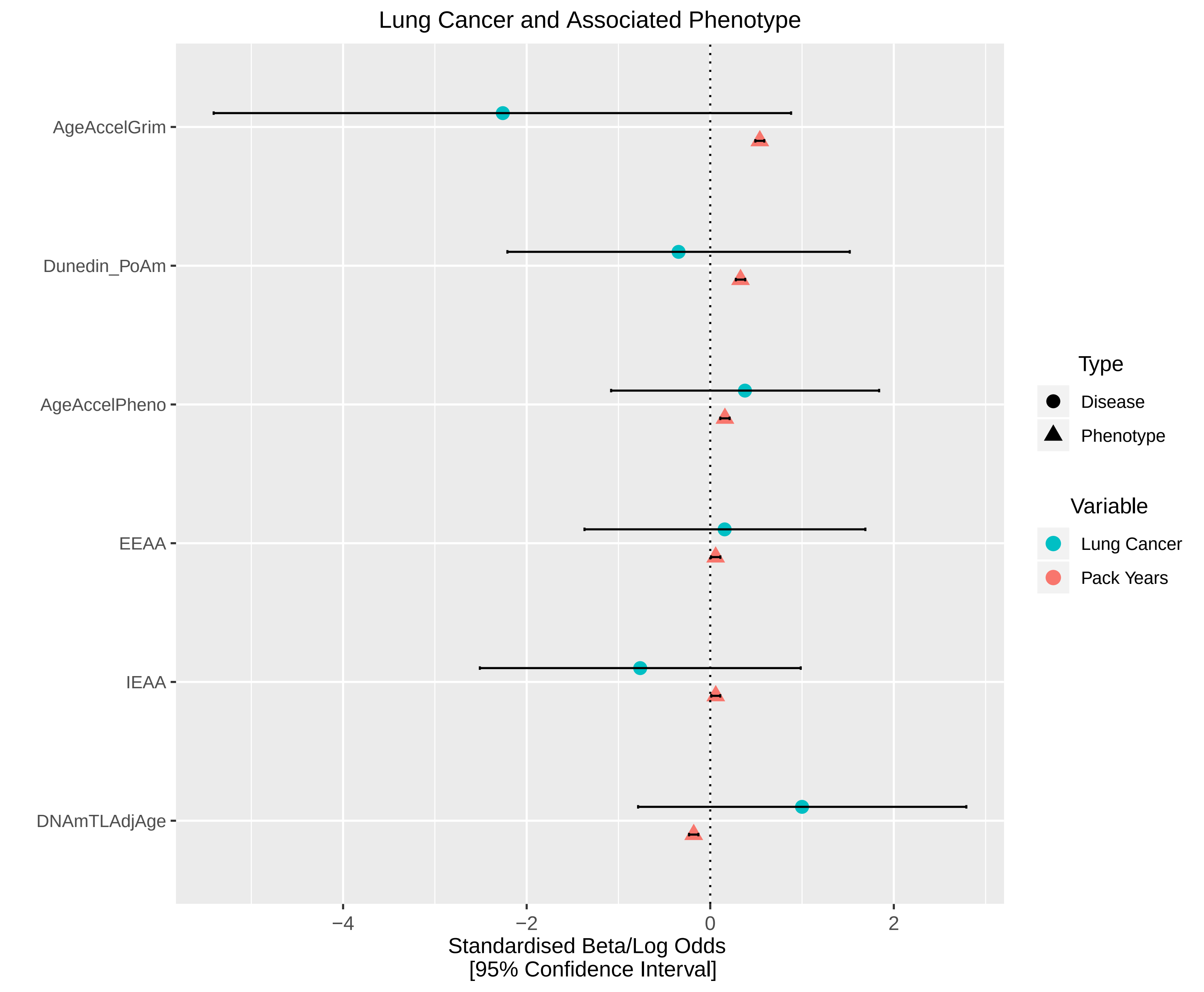
Lung Cancer and Associated Phenotype
AgeAccelGrim
Dunedin_PoAm
AgeAccelPheno
EEAA
IEAA
DNAmTLAdjAge
−4
−2
0
2
Standardised Beta/Log Odds
[95% Confidence Interval]
Type
Disease
Phenotype
Variable
Lung Cancer
Pack Years

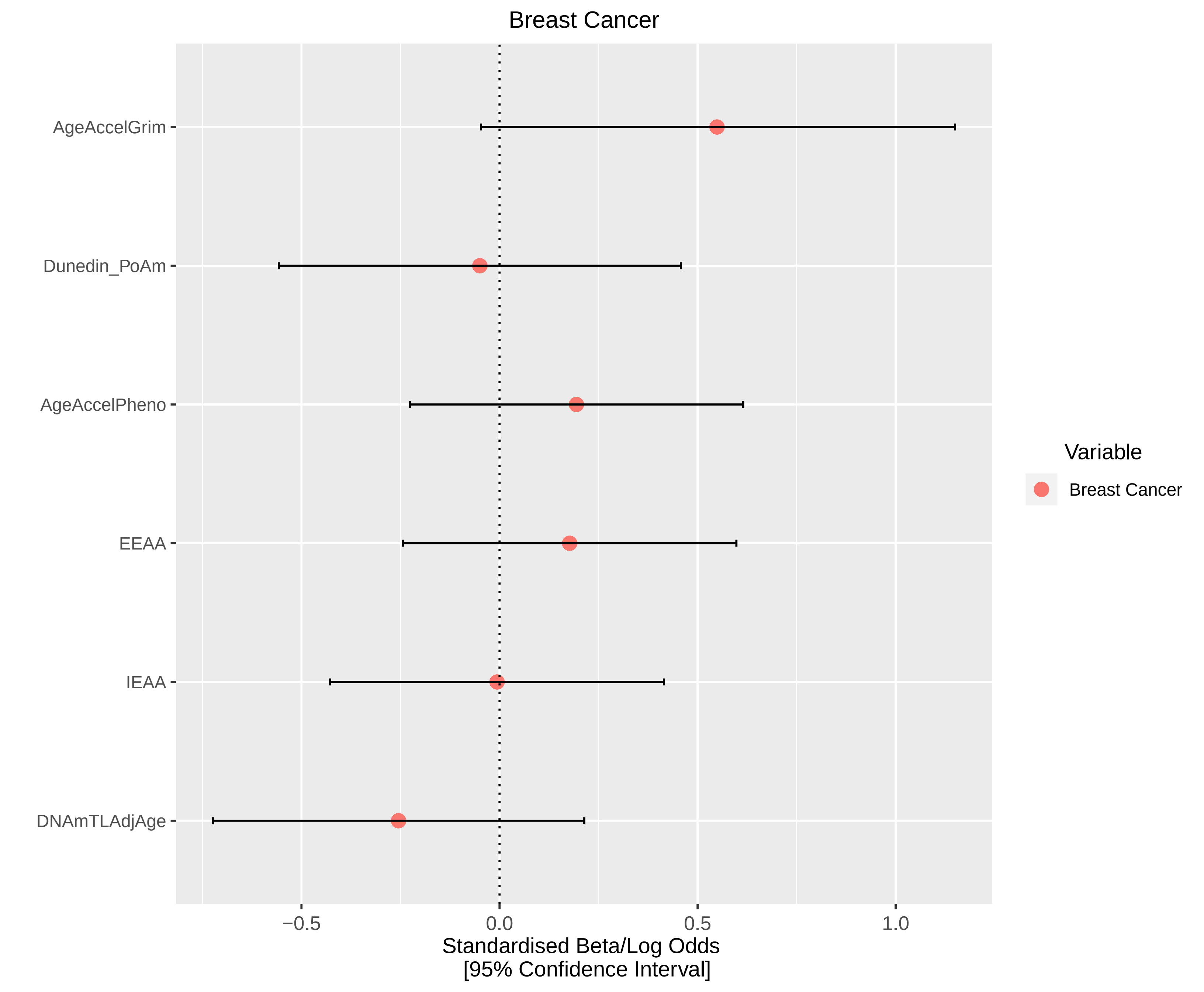
Breast Cancer
AgeAccelGrim
Dunedin_PoAm
AgeAccelPheno
EEAA
IEAA
DNAmTLAdjAge
−0.5
0.0
0.5
1.0
Standardised Beta/Log Odds
[95% Confidence Interval]
Variable
Breast Cancer

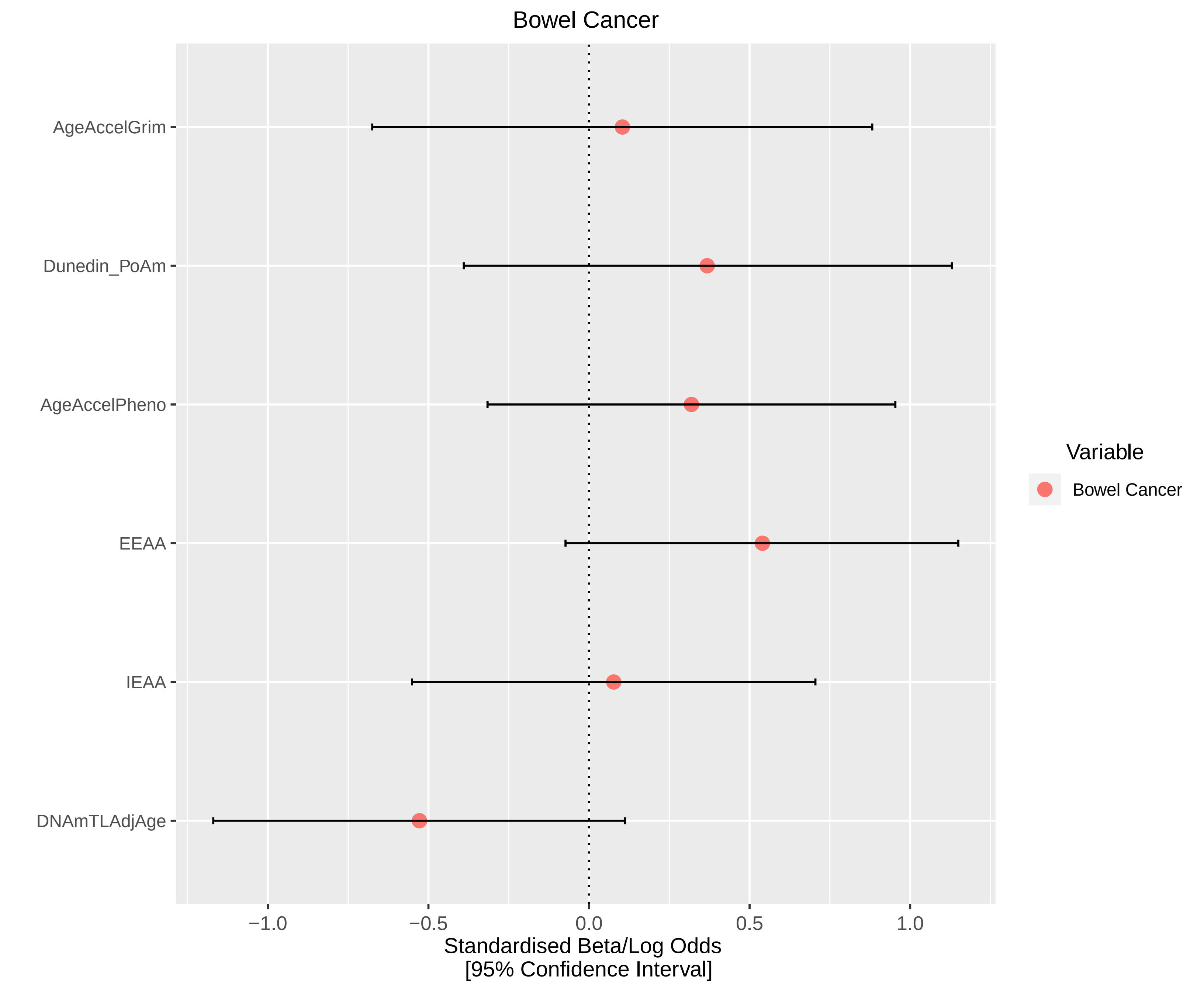
Bowel Cancer
AgeAccelGrim
Dunedin_PoAm
AgeAccelPheno
EEAA
IEAA
DNAmTLAdjAge
−1.0
−0.5
0.0
0.5
1.0
Standardised Beta/Log Odds
[95% Confidence Interval]
Variable
Bowel Cancer

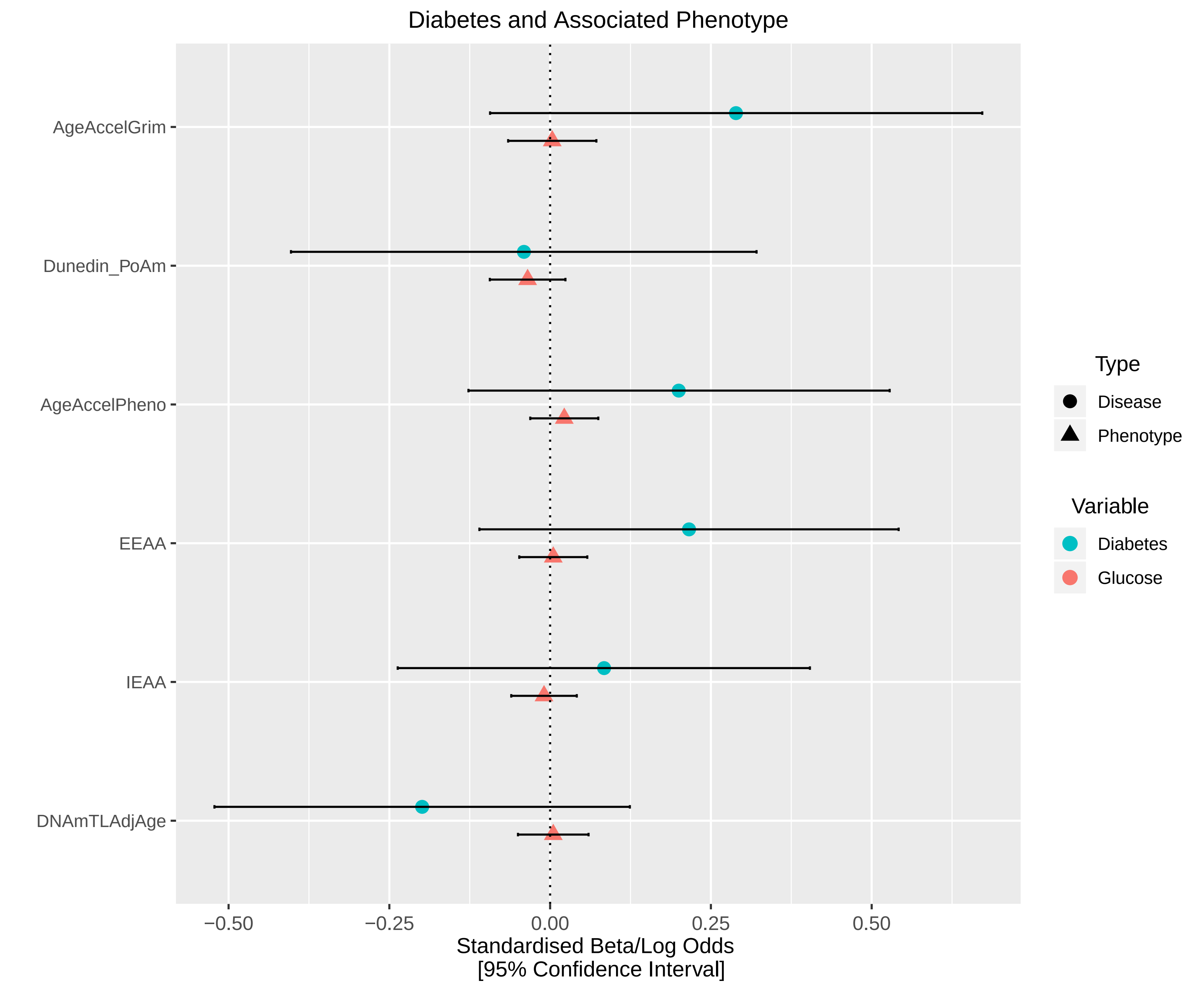
Diabetes and Associated Phenotype
AgeAccelGrim
Dunedin_PoAm
AgeAccelPheno
EEAA
IEAA
DNAmTLAdjAge
−0.50
−0.25
0.00
0.25
0.50
Standardised Beta/Log Odds
[95% Confidence Interval]
Type
Disease
Phenotype
Variable
Diabetes
Glucose

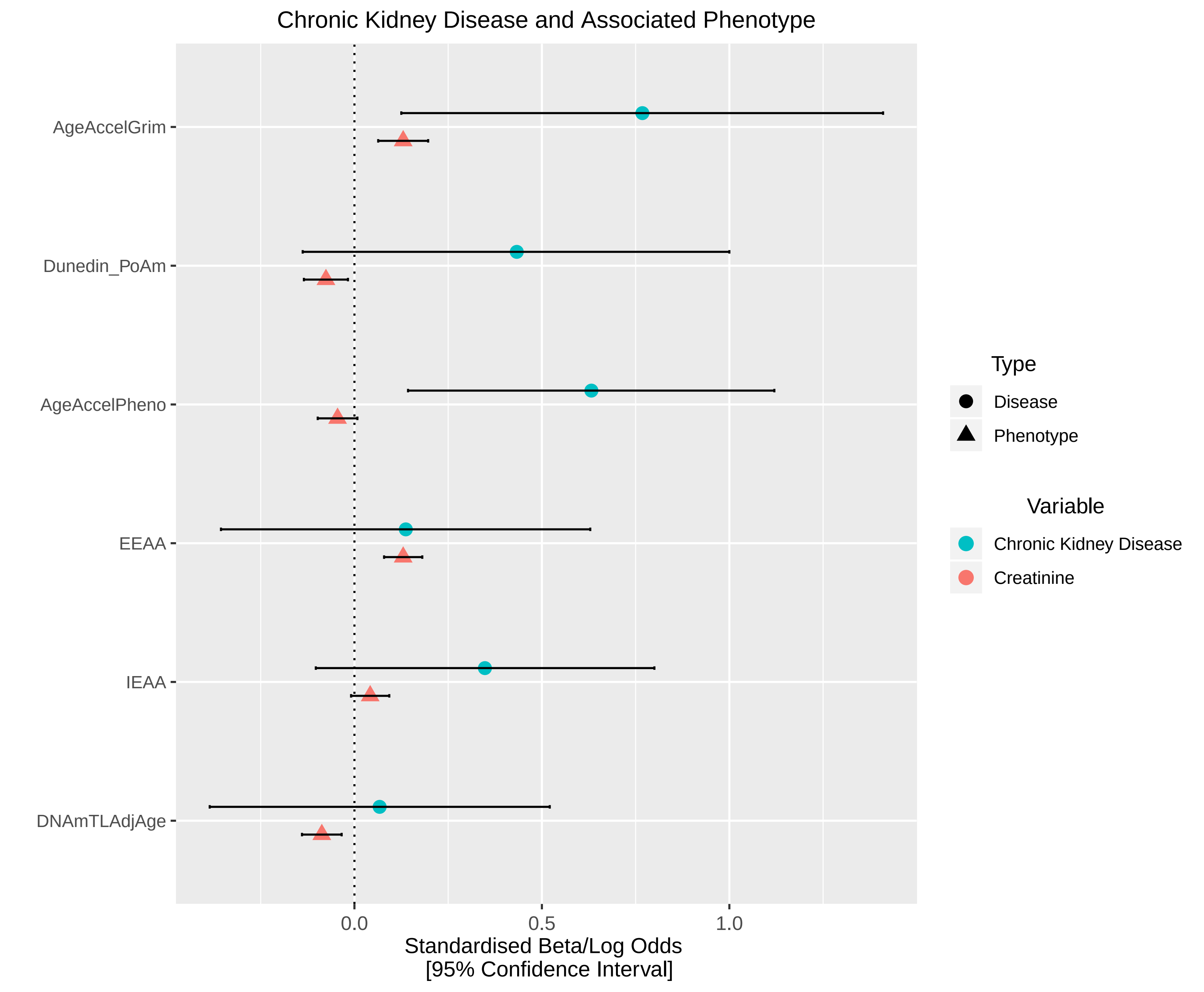

Chronic Kidney Disease and Associated Phenotype
AgeAccelGrim
Dunedin_PoAm
AgeAccelPheno
EEAA
IEAA
DNAmTLAdjAge
0.0
0.5
1.0
Standardised Beta/Log Odds
[95% Confidence Interval]
Type
Disease
Phenotype
Variable
Chronic Kidney Disease
Creatinine

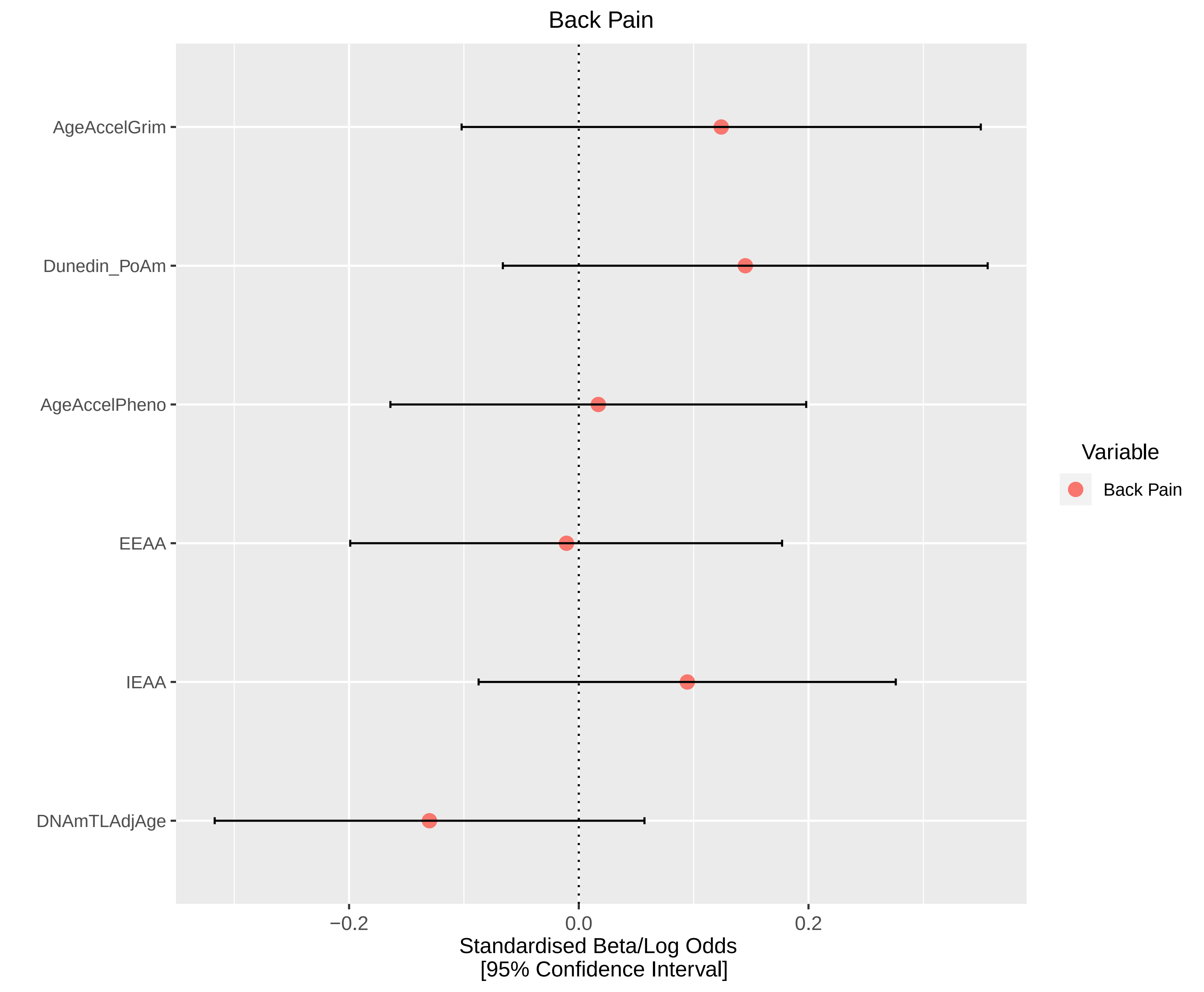
Back Pain
AgeAccelGrim
Dunedin_PoAm
AgeAccelPheno
EEAA
IEAA
DNAmTLAdjAge
−0.2
0.0
0.2
Standardised Beta/Log Odds
[95% Confidence Interval]
Variable
Back Pain

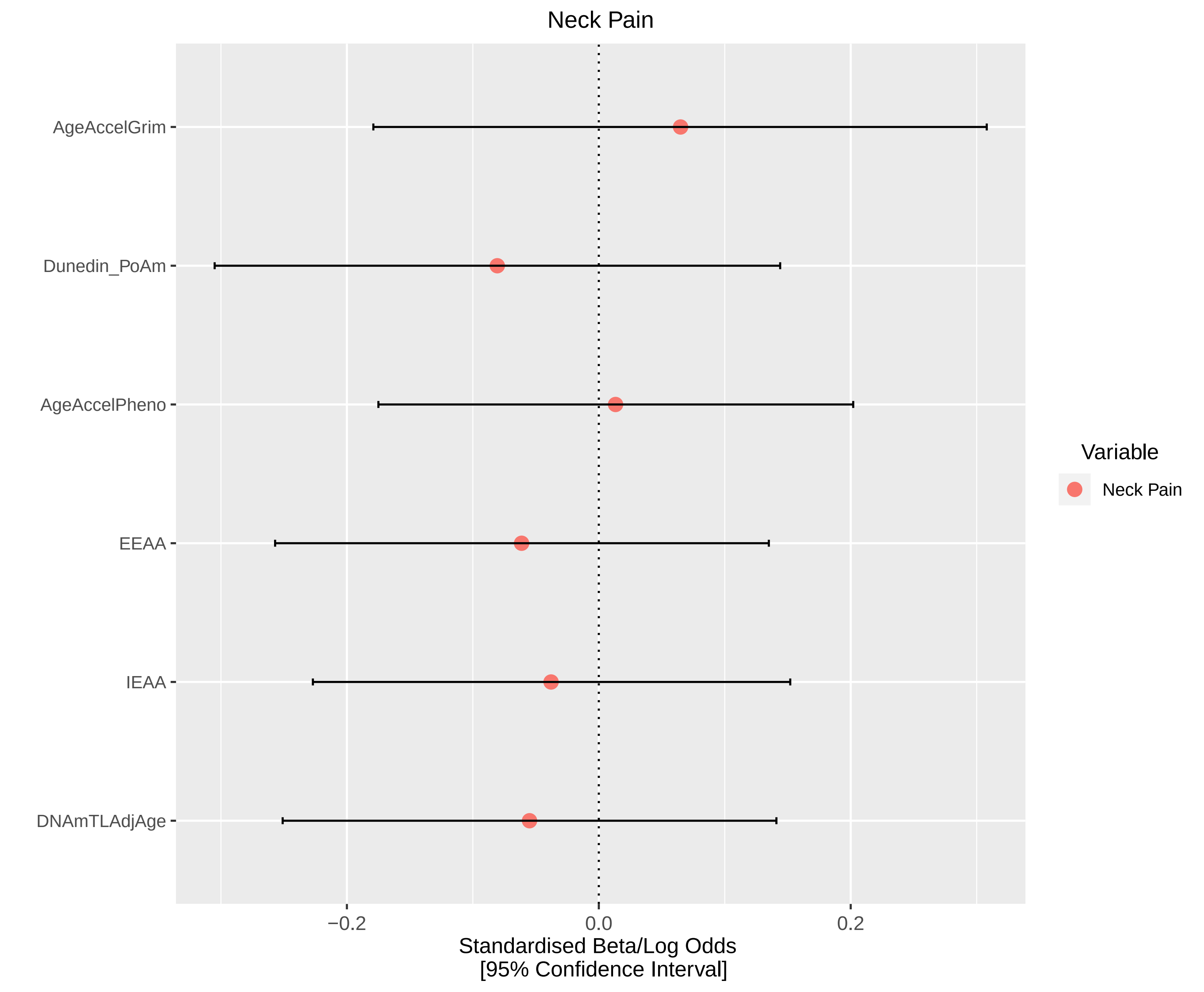

Neck Pain
AgeAccelGrim
Dunedin_PoAm
AgeAccelPheno
EEAA
IEAA
DNAmTLAdjAge
−0.2
0.0
0.2
Standardised Beta/Log Odds
[95% Confidence Interval]
Variable
Neck Pain
