# Supplementary material for: Epigenetic measures of ageing predict the prevalence and incidence of leading causes of death and disease burden
Source: Clin Epigenetics. 2020 Jul 31;12:115. doi: 10.1186/s13148-020-00905-6 (PMC7394682; doi:10.1186/s13148-020-00905-6)
Supplement: Supplementary file 6 — Additional file 6. Supplementary Note 2. Associations between epigenetic measures of ageing and incidence of ICD-10-coded common diseases in a basic model adjusting for age and sex. [file 13148_2020_905_MOESM6_ESM.docx]

**Additional file 6 - Supplementary Note 2. Associations between epigenetic measures of ageing and incidence of ICD-10-coded common diseases in a basic model adjusting for age and sex.**

*DNAm GrimAge*

AgeAccelGrim was associated with incidence of COPD (Hazard Ratio (HR) per SD = 2.65, P = 1.2 x 10^-58^), diabetes (HR = 1.75, P = 9.6 x 10^-15^), ischemic heart disease (HR = 1.46, P = 1.4 x 10^-13^), stroke (HR = 1.43, P = 1.8 x 10^-6^) and lung cancer (HR = 1.46, P = 6.5 x 10^-11^). AgeAccelGrim also showed a nominally significant association with incidence of depression (HR = 1.42, P = 0.01), Alzheimer’s disease (HR = 1.70, P = 0.03) and dorsalgia (HR = 1.22, P = 0.04).

*DunedinPoAm*

DunedinPoAm was associated with incidence of COPD (HR = 2.62, P = 2.5 x 10^-40^), diabetes (HR = 1.64, P = 9.0 x 10^-10^), stroke (HR = 1.52, P = 8.1 x 10^-8^), lung cancer (HR = 1.47, P = 3.6 x 10^-8^) and ischemic heart disease (HR = 1.31, P = 2.9 x 10^-6^). DunedinPoAm also showed a nominally significant association with incidence of depression (HR = 1.42, P = 0.02), Alzheimer’s disease (HR = 1.67, P = 0.03) and dorsalgia (HR = 1.30, P = 0.04).

*DNAm PhenoAge*

AgeAccelPheno was associated with incidence of diabetes (HR = 1.73, P = 8.0 x 10^-14^), COPD (HR = 1.58, P = 8.0 x 10^-11^) and ischemic heart disease (HR = 1.29, P = 9.6 x 10^-7^). AgeAccelPheno showed nominally significant associations with incidence of depression (HR = 1.37, P = 0.03), stroke (HR = 1.18, P = 0.02) and breast cancer (HR = 1.36, P = 0.01).

*HannumAge*

Age-adjusted HannumAge (EEAA) was nominally associated with incidence of COPD (HR = 1.25, P = 1.1 x 10^-3^).

*HorvathAge*

Age-adjusted HorvathAge (IEAA) showed a nominally significant relationship with incidence of diabetes (HR = 1.18, P = 0.03).

*DNAmTLadjAge*

Age-adjusted DNAm Telomere Length was associated with incidence of COPD (HR = 0.64, P = 2.8 x 10^-10^), ischemic heart disease (HR = 0.81, P = 7.5 x 10^-5^), stroke (HR = 0.75, P = 1.3 x 10^-4^) and diabetes (HR = 0.76, P = 8.0 x 10^-4^), and also showed a nominally significant association with incidence of depression after 13 years since study baseline (HR = 0.71, P = 0.02).

Please refer to Additional File 3: Table S9 for full details of association models.
