# Supplementary material for: Epigenetic measures of ageing predict the prevalence and incidence of leading causes of death and disease burden
Source: Clin Epigenetics. 2020 Jul 31;12:115. doi: 10.1186/s13148-020-00905-6 (PMC7394682; doi:10.1186/s13148-020-00905-6)
Supplement: Supplementary file 7 — Additional file 7. Details of Supplementary Methods. [file 13148_2020_905_MOESM7_ESM.docx]

**Additional file 7 - Supplementary Methods**

DNA methylation (DNAm) levels were measured using the Illumina HumanMethylationEPIC BeadChip Array on blood samples from Generation Scotland participants. Outliers were identified and excluded through visually inspecting a plot of log median intensity of methylated versus unmethylated signal. Samples were excluded in instances where ≥1% of CpG sites had a detection P-value in excess of 0.05, and where recorded sex did not match DNAm-predicted sex. Probes were removed in instances where ≥ 0.5% of samples had a detection p-value in excess of 0.05, and where there were beadcounts of less than 3 in 6% or more samples.

To derive measures for five of the epigenetic measures (excluding DunedinPoAm), we applied an additional quality control approach to those samples that had passed the screening process as described above. This was carried out to eliminate missing CpG values, as recommended in Horvath’s epigenetic clock tutorial (https://dnamage.genetics.ucla.edu/). Raw methylation IDAT files were read into *R* using the *minfi* package. Data were normalised through the normal-exponential convolution using out-of-band probe (noob) method implemented by the *preprocessNoob*() function in *minfi*. Briefly, this method consists of a background-subtraction step and dye-bias normalisation. Background noise is estimated from out-of-band probes and is removed from each sample whereas dye-bias normalisation utilises a subset of control probes to estimate the dye-bias. Noob-normalised methylation beta values were then obtained using the *getBeta*() function in *minfi*. Measures of DunedinPoAm were calculated using methylation betas from the EPIC array and an *R* package created by the original study’s authors which is available on the following github repository: https://github.com/danbelsky/DunedinPoAm38.

***Horvath Age***

Horvath Age gives rise to a measure of biological ageing termed “intrinsic epigenetic age acceleration (IEAA)” as it is independent of age-related changes in blood composition (1). An elastic net regression model was used to regress chronological age onto 21369 probes that were present on both the Illumina 450k and 27k platforms and possessed fewer than 10 missing values. Penalised regression selected 353 CpGs as informative for predicting chronological age. The linear combination of these CpG sites provides an estimate of DNAm Horvath Age. Subsequently, IEAA was obtained as the residual term from regressing Horvath Age onto chronological age and fitting counts of naive CD8^+^ T-cells, exhausted CD8^+^ T-cells, plasmablasts, CD4^+^ T-cells, natural killer cells, monocytes, and granulocytes estimated from the methylation data.

***Hannum Age***

Hannum age produces a measure of epigenetic ageing referred to as “extrinsic epigenetic age acceleration (EEAA)” as it tracks age-related changes in blood cell composition (2). Elastic net regression identified 71 CpGs from the Illumina 450K platform as informative for predicting chronological age. EEAA was calculated in a two-step process. Firstly, a weighted average was calculated from Hannum Age and three cell types whose abundance exhibits age-related changes (naive cytotoxic T-cells, exhausted cytotoxic T-cells, and plasmablasts) (3). Secondly, EEAA was obtained by regressing this weighted average onto chronological age.

***DNAm PhenoAge***

DNAm PhenoAge was developed in a two-stage process by Levine *et al.* (4). In the first step, a novel indicator of biological age, termed ‘Phenotypic Age’, was derived from the employment of an elastic net regression model in which the hazard of mortality was regressed onto 42 markers from the third National Health and Nutrition Examination Survey. In addition to chronological age, the model selected 9 haematological and biochemical markers for inclusion in the ‘Phenotypic Age’ predictor. These markers were: albumin, alkaline phosphatase, creatinine, C-reactive protein (CRP), mean cell volume, percentage lymphocytes, red cell distribution width, serum glucose (as indexed by glycated HbA1c) and white blood cell count. In the second stage, a second elastic net model was used to regress ‘Phenotypic Age’ onto 20169 CpG sites (which represent those CpG sites present on all Illumina arrays i.e. 27K, 450K and EPIC). The model selected 513 CpG sites, the linear combination of which gives rise to a methylation-based surrogate of ‘Phenotypic Age’ termed ‘DNAm PhenoAge’. Importantly, the residual term from regressing DNAm PhenoAge onto chronological age produces a measure of biological age acceleration termed ‘AgeAccelPheno’.

**DNAm GrimAge**

In contrast to other clocks, which use chronological age as the dependent variable in elastic net regression models, Lu *et al.* developed a novel clock termed ‘DNAm GrimAge’ which uses mortality as a reference (5). They firstly created DNAm-based surrogates of smoking pack years and 88 plasma protein levels. DNAm-based surrogates of protein levels were required to exhibit a moderate correlation coefficient of > 0.35 with the respective phenotype to be considered for stage two. Only 12 out of 88 proteins satisfied this condition. Consequently, for the second stage, an elastic net Cox regression model was used to regress time to death due to all-cause mortality onto chronological age, sex and methylation-based surrogates for smoking pack years and 12/88 proteins. The model selected chronological age, sex, DNAm smoking pack years and DNAm surrogates for 7/12 protein levels: adrenomedulin (DNAm ADM), beta-2-microglobulin (DNAm B2M), cystatin C (DNAm Cystatin C), growth differentiation factor 15 (DNAM GDF-15), leptin (DNAm leptin), plasminogen activation inhibitor 1 (DNAm PAI-1) and tissue inhibitor metalloproteinaise (DNAm TIMP-1). DNAm-based surrogates for smoking pack years and the 7 plasma proteins totalled 1030 unique CpG sites from those present on both the Illumina 450K and EPIC array platforms. The linear combination of these variables provides an estimate of an individual’s DNAm GrimAge which correlates strongly with risk of mortality. The residual term from regressing DNAm GrimAge onto chronological age provides an index of biological age acceleration with respect to mortality risk and is termed ‘AgeAccelGrim’.

***DNAm Telomere Length***

Lu *et al.* developed an epigenetic correlate of telomere length in leukocytes, which is known to associate with a variety of age-related conditions. This biomarker termed ‘DNAm Telomere Length’ or ‘DNAm TL’ was derived using an elastic net regression model (6). In a similar design to DNAm GrimAge, CpG sites present on the Illumina 450K and Illumina EPIC Array platforms were regressed onto leukocyte telomere length. The model selected 140 CpG sites, and the weighted linear combination of methylation values at these CpG sites produced an individual’s estimate of DNAm TL. Interestingly, DNAm TL exhibited a higher correlation with age than measured leukocyte telomere length and outperformed its phenotypic counterpart in predicting time-to-death, time-to-coronary heart disease and time-to-congestive heart failure. A novel index of biological ageing was then derived by regressing DNAm TL onto chronological age which was termed ‘DNAmTLadjAge’. In contrast to other clocks, a lower age-adjusted DNAm TL is anticipated to correlate with poorer health outcomes as this reflects the age-related process of cellular telomere shortening.

***DunedinPoAm***

The five epigenetic clocks described above are all derived from examining *cross-sectional* associations between DNAm profiles and the outcome of interests: chronological age, biological age, telomere length or mortality risk. In contrast, the Dunedin ‘Pace of Aging’ (PoA) measure was trained on *longitudinal* changes in 18 blood-chemistry and organ-system-function biomarkers at three distinct time points from age 26 to 38 years (n = 964 individuals) (7). This measure exploits inter-individual variation in the rate of biological ageing in individuals who were born within the same year, thus making it a speedometer when compared to the existing epigenetic clocks. The previous clocks were also trained on individuals born within different years in an effort to track pseudo-longitudinal trajectories of ageing. However, this is confounded by the possibility that individuals who were born within different timeframes may have been exposed to different early-life environmental conditions, such as higher levels of tobacco smoke and airborne lead (8). This may result in individuals displaying differential DNAm profiles that are not related to ageing but rather related to contrasting early-life exposures. The 18 biomarkers used to build the Dunedin PoA measure were: HbA1c, cardiorespiratory fitness, waist-hip ratio, FEV_1_/FVC ratio, FEV_1_, mean arterial pressure, body mass index, leukocyte telomere length, creatinine clearance, blood urea nitrogen, lipoprotein (a), triglycerides, gum health, total cholesterol, white blood cell count, high-sensitivity C-reactive protein, HDL cholesterol, ApoB100/ApoA1 ratio. Three of these biomarkers overlap with those used in the derivation of DNAm PhenoAge: HbA1c, C-reactive protein and white blood cell count. The biomarkers were included to reflect aspects of the integrity of cardiovascular, metabolic, renal, hepatic, pulmonary, periodontal, and immune systems. For each biomarker, mixed-effects growth modelling was used to calculate the rate of change (slope) for all individuals incorporating data points from age 26, 32 and 38 years. Each individual’s PoA was calculated as a sum of these random slopes. To proxy this PoA measure, Belsky *et al.* (2020) built on their previous work by employing elastic net regression to derive a DNAm-based predictor of the PoA measure (9). PoA scores for Dunedin study participants at age 38 were regressed onto DNAm data obtained at the same time point. This created an epigenetic ageing measure termed ‘DunedinPoAm’. This measure of the pace of biological ageing is derived from 46 unique CpG sites and can be derived from either Illumina 450K or EPIC array data through an *R* package available from github (https://github.com/danbelsky/DunedinPoAm38). The authors tested the validity of the DunedinPoAm measure in five ways. First, they showed that DunedinPoAm calculated at age 38 in Dunedin study participants was associated with poorer physical and cognitive function during follow-up at age 45. Second, using data from the cross-sectional Understanding Society study, the authors characterised relationships between DunedinPoAm and chronological age as well as sex (https://www.understandingsociety.ac.uk/). They also provided correlations between DunedinPoAm and self-reported health as well as Horvath Age, Hannum Age and DNAm PhenoAge. Third, the authors showed that DunedinPoAm associated with chronic-disease morbidity and mortality in the Normative Aging Study cohort which is a longitudinal study of ageing in older men (10). Fourth, DunedinPoAm was calculated for participants of the Environmental Risk (E-Risk) study, which is a longitudinal study of younger individuals, and showed that this measure of biological ageing associated with exposure to poverty and victimisation which may shorten healthy lifespan (11). Fifth, the authors tested whether DunedinPoAm was altered in individuals who underwent 2 years of prescribed 25% caloric restriction in the CALERIE trial when compared to controls (12). However, there was no statistically significant difference between the rate of ageing for individuals in the control and treatment arms of the trial, as captured by DunedinPoAm.

**References**

1. Horvath S. DNA methylation age of human tissues and cell types. Genome biology. 2013;14(10):R115.

2. Hannum G, Guinney J, Zhao L, Zhang L, Hughes G, Sadda S, et al. Genome-wide methylation profiles reveal quantitative views of human aging rates. Molecular cell. 2013;49(2):359-67.

3. Klemera P, Doubal S. A new approach to the concept and computation of biological age. Mechanisms of ageing and development. 2006;127(3):240-8.

4. Levine ME, Lu AT, Quach A, Chen BH, Assimes TL, Bandinelli S, et al. An epigenetic biomarker of aging for lifespan and healthspan. Aging. 2018;10(4):573-91.

5. Lu AT, Quach A, Wilson JG, Reiner AP, Aviv A, Raj K, et al. DNA methylation GrimAge strongly predicts lifespan and healthspan. Aging (Albany NY). 2019;11(2):303-27.

6. Lu AT, Seeboth A, Tsai PC, Sun D, Quach A, Reiner AP, et al. DNA methylation-based estimator of telomere length. Aging (Albany NY). 2019.

7. Belsky DW, Caspi A, Houts R, Cohen HJ, Corcoran DL, Danese A, et al. Quantification of biological aging in young adults. Proceedings of the National Academy of Sciences of the United States of America. 2015;112(30):E4104-E10.

8. Bell CG, Lowe R, Adams PD, Baccarelli AA, Beck S, Bell JT, et al. DNA methylation aging clocks: challenges and recommendations. Genome biology. 2019;20(1):249.

9. Belsky DW, Caspi A, Arseneault L, Baccarelli A, Corcoran DL, Gao X, et al. Quantification of the pace of biological aging in humans through a blood test, the DunedinPoAm DNA methylation algorithm. eLife. 2020;9.

10. Bell B, Rose CL, Damon A. The Normative Aging Study: An Interdisciplinary and Longitudinal Study of Health and Aging. Aging and Human Development. 1972;3(1):5-17.

11. Moffitt TE. Teen-aged mothers in contemporary Britain. Journal of child psychology and psychiatry, and allied disciplines. 2002;43(6):727-42.

12. Ravussin E, Redman LM, Rochon J, Das SK, Fontana L, Kraus WE, et al. A 2-Year Randomized Controlled Trial of Human Caloric Restriction: Feasibility and Effects on Predictors of Health Span and Longevity. J Gerontol A Biol Sci Med Sci. 2015;70(9):1097-104.
